# A balance score between immune stimulatory and suppressive microenvironments identifies mediators of tumour immunity and predicts pan-cancer survival

**DOI:** 10.1038/s41416-020-01145-4

**Published:** 2020-11-05

**Authors:** Tolga Turan, Sarah Kongpachith, Kyle Halliwill, Jessica Roelands, Wouter Hendrickx, Francesco M. Marincola, Thomas J. Hudson, Howard J. Jacob, Davide Bedognetti, Josue Samayoa, Michele Ceccarelli

**Affiliations:** 1 Computational Immunology and Oncology (CIAO), AbbVie, Redwood City, CA USA; 2 Cancer Research Department, Sidra Medicine, Doha, Qatar; 3 Department of Surgery, Leiden University Medical Center (LUMC), Leiden, The Netherlands; 4 Refuge Biotechnologies, Menlo Park, CA USA; 5 Genomics Research Center (GRC), AbbVie, Lake County, IL USA; 6 Dipartimento di Medicina Interna e Specialità Mediche, Università degli Studi di Genova, Genova, Italy; 7 Department of Electrical Engineering and Information Technology, University of Naples “Federico II”, Naples, Italy; 8 Istituto di Ricerche Genetiche “G. Salvatore”, Biogem s.c.ar.l, 83031 Ariano Irpino, Italy

**Keywords:** Classification and taxonomy, Cancer genomics, Immunotherapy, Bioinformatics

## Abstract

**Background:**

The balance between immune-stimulatory and immune-suppressive mechanisms in the tumour microenvironment is associated with tumour rejection and can predict the efficacy of immune checkpoint-inhibition therapies.

**Methods:**

We consider the observed differences between the transcriptional programmes associated with cancer types where the levels of immune infiltration predict a favourable prognosis versus those in which the immune infiltration predicts an unfavourable prognosis and defined a score named **M**ediators of **I**mmune **R**esponse **A**gainst **C**ancer in so**L**id micro**E**nvironments (MIRACLE). MIRACLE deconvolves T cell infiltration, from inhibitory mechanisms, such as TGFβ, EMT and PI3Kγ signatures.

**Results:**

Our score outperforms current state-of-the-art immune signatures as a predictive marker of survival in TCGA (*n* = 9305, HR: 0.043, *p* value: 6.7 × 10^−36^). In a validation cohort (*n* = 7623), MIRACLE predicts better survival compared to other immune metrics (HR: 0.1985, *p* value: 2.73 × 10^−38^). MIRACLE also predicts response to checkpoint-inhibitor therapies (*n* = 333). The tumour-intrinsic factors inversely associated with the reported score such as EGFR, PRKAR1A and MAP3K1 are frequently associated with immune-suppressive phenotypes.

**Conclusions:**

The association of cancer outcome with the level of infiltrating immune cells is mediated by the balance of activatory and suppressive factors. MIRACLE accounts for this balance and predicts favourable cancer outcomes.

## Background

The presence of an active immune microenvironment with a high density of activated T cells associates with favourable prognosis and responsiveness to immunotherapy.^1^ Genetic factors preventing the development of a favourable immune milieu and/or responsiveness to immunotherapy include limited expression of neoantigens,^2,3^ which is partially a function of mutational load, and high tumour aneuploidy. However, in the “immune-active” tumours, upregulation of pro-inflammatory signals is accompanied by the expression of interferon-gamma-inducible immune regulatory molecules like programmed death ligand-1 (PD-L1) and indoleamine 2,3-dioxygenase dioxygenase (IDO), whose levels correlate with response to anti-PD-1 and anti-cytotoxic T-lymphocyte-associated protein 4 (anti-CTLA4) treatment. The expression of such molecules as well as of other immune-regulatory markers (e.g., FOXP3, CTLA4 and PD-1) characterise a *compensatory immune resistance*, reflecting the presence of counter-regulatory mechanisms that follow, rather than precede,^4,5^ the recognition of tumour antigens by T cells and the subsequent amplification of the inflammatory response.^6,7^ Correlative studies in humans and experimental models suggest that checkpoint inhibitors are less effective in tumours characterised by a primary immune suppression (also called as “primary immune ignorance”), including the ones with low mutational load,^1,8^ or dominated by the genomic dysregulations of oncogenic pathways leading to T cell exclusion such as WNT/beta-catenin,^9–11^ mitogen-activated protein kinase^6,7,12^ and transforming growth factor-β (TGFβ) pathways^13–15^ The dichotomy between “immune active” (associated with the displaying of compensatory immune resistance) and “immune silent” (typified by the presence of a primary immune suppression) might be useful to explain a general phenomenon but do not reflect the high level of inter-patient heterogeneity and do not take into account the contribution of antagonist signals involved in primary immune suppression. In fact, one of the major limitations of the transcriptomic studies performed so far is the use of gene signatures or modules that capture only a dominant process or a group of processes tightly interconnected or correlated among each other.

We speculate that the concurrent measurement of stimulatory and suppressive immunologic signals might better define the true immune state of the tumour–immune microenvironment and thus develop the **M**ediators of **I**mmune **R**esponse **A**gainst **C**ancer in so**L**id micro**E**nvironments (MIRACLE), a score that captures the balance between opposite immune-related signals.

We show that MIRACLE is a better prognostic factor than the current immune-associated signatures. We also evaluate MIRACLE in the context of response to immune checkpoint inhibitor (ICI) therapy. Further, in contrast to other measures of the tumour–immune microenvironment,^16,17^ MIRACLE is an absolute measure that can be applied to a single sample and does not need any data set-specific normalisation.

## Methods

### Data collection

All The Cancer Genome Atlas (TCGA) data (31 solid tumour cohorts) were downloaded using the TCGAbiolinks package and from Genomic Data Commons.^18^ RNAseq expression data were downloaded in the form of raw counts and survival attributes were downloaded in the form of Clinical Data Tables. All cohorts except TCGA-SKCM were exclusively primary tumours. Cancer types, Pheochromocytoma and Paraganglioma (PCPG), Thymoma (THYM) and Testicular Germ cell tumours (TGCT), were excluded from the analysis as the number of deaths in the comparison groups was too small for survival estimation.^19^

Besides TCGA, publicly available data sets were downloaded from NCBI Gene Expression Omnibus (GEO), European Nucleotide Archive or Short Read Archive. The clinical phenotypes were harmonised between raw or processed data and the associated publications. GEO data sets (or data sets from other Public Repositories) were treated as separate studies and were not merged, even when they are annotated as the same Solid Tumour Cohort.

For microarray data sets, processed data with acceptable normalisation (quantile, RMA or equivalent) were downloaded as series matrix files from GEO, when available. If processed data were not available, raw data were downloaded. For RNAseq data sets, raw gene counts were downloaded when available, otherwise raw.fastq files were downloaded and processed (Table S1 lists the public data sets used in this study.).

### Data processing

All data processing was done in R version 3.5.2 except when indicated.

#### RNA sequencing

Raw fastq files were adapter- and quality-trimmed using Trim_Galore/CutAdapt version 3.5.2. The trimmed reads were aligned to hg19 using STAR RNAseq aligner (STAR_2.5.3a). Raw gene counts were generated using summarizeOverlaps functions of the GenomicAlignments Bioconductor package. Raw counts were then normalised (within lane and between lane) using EDASeq Bioconductor package. All heatmaps and geneset enrichment analyses were performed on the EDAseq normalised matrices.

#### Microarray

Affymetrix.CEL files were downloaded from GEO using “GEOquery” Bioconductor package and normalised using justRMA function of GEOquery.

#### Geneset enrichment

For single sample gene set enrichment, we used the yaGST package.^20^

Precalculated TCGA “Immune Landscape of Cancer” scores were downloaded from the respective publication.^13^ The gene signatures used to calculate remaining enrichment scores can be found in Supplementary Table S2.^5^ ESTIMATE-immune and -stromal signatures were downloaded from the respective publication.^21^ The signatures from these sources (83 signatures/predictors) are further filtered due to missing data or outlier behaviour. A total of 76 signatures were used to benchmark against MIRACLE within 28 solid TCGA cohorts in survival and correlation analyses.

### Stratification of cohorts based on immunologic constant of rejection (ICR)–survival association

Normalised enrichment scores (NESs) for ICR gene signature were used as a continuous metric to identify survival association in 28 solid TCGA cohorts individually. The cohorts with positive survival association are designated as ICR-Enabled (IE; hazard ratio (HR) < 1 with a *p* value < 0.1) and the cohorts with significant negative survival association are designated as ICR-Disabled (ID) cohorts (HR > 1 with a *p* value < 0.1)

### Differential expression

To identify the genomic features correlated with ICR in IE and ID cohorts, we performed supervised differential expression analysis between samples with high enrichment of ICR genes versus samples with low enrichment of ICR, pooling together all the samples in each cohort (IE and ID). High and low enrichment levels were defined according to the first and third tertile of the NES. We used the non-parametric Wilcoxon test (R function wilcox.test) and filtered the genes with log2 Fold Change > 1, *p* value < 0.05 and adjusted *p* value < 0.1; these genes constitute the IE and ID genesets (Supplementary Table S3).

### Computation of the MIRACLE score

MIRACLE is the ratio between the single-sample enrichment scores of two gene sets, the top genes of Δ*R*_IE_ and Δ*R*_ID_ in a given sample. We used the NES of the Mann–Whitney–Wilcoxon Gene Set test (mww-GST) available in the yaGST package.^20^ Briefly, NES is an estimate of the probability that the expression of a gene in the geneset is greater than the expression of a gene outside this set:

$${\rm{NES}} = 1 - \frac{U}{{mn}},$$

where *m* is the number of genes in a gene set, *n* is the number of those outside the gene set, *U* = *mn* + *m*(*m* + 1) − *T* and *T* is the sum of the ranks of the genes in the geneset.

### Gene ontology (GO) and network analysis

GO analyses were performed using the BiNGO application in the Cytoscape Suite version 3.7.1. GO enrichments are calculated starting from top IE genes (*n* = 605) and ID genes (*n* = 377) using the hypergeometric test. To visualise the enriched GO terms in a network graph, the Cytoscape EnrichmentMap application was used. To exclude very general or too granular GO terms, BiNGO output was filtered removing GO terms corresponding to gene sets with >600 features or <10 features. In the network images (EnrichmentMap), the size of the nodes is proportional to the geneset size while edge thickness is proportional to the relatedness of the GO terms (Jaccard similarity coefficient).

### Survival analysis

To calculate survival statistics for continuous or categorical variables, coxph function from the survival R package was used. When testing the predictive value of a signature in a single cohort, univariate analysis is used. In pan-cancer analysis of multiple cohorts, “Cohort” variable is added to the model (Survival ~ Predictor + Cohort). In multivariate analysis, variables “Age”, “Gender” and “Stage” are added to the model. To compare different models and additive value of each variable, analysis of variance method was used.

Model 1: Survival ~ Predictor + Cohort

Model 2: Survival ~ Predictor + Cohort + Age + Gender + Stage

Model 3: Survival ~ Cohort + Age + Gender + Stage

When testing large number (*n* > 10) of survival models (i.e. multiple cohorts for a single predictor or multiple predictors for single cohort), adjusted *p* values are used (Benjamin–Hochberg).

### ICI response definitions and data sets

Any patient classified as “Progressive Disease” based on RECIST criteria was considered as a “Non-Responder”. Any patient classified as “Complete Response”, “Partial Response” or “Stable Disease” was considered as a “Responder”. Receiver operating characteristic (ROC) curves and area under the curve (AUC) values are calculated using “pROC” R package based on above response definitions and associated MIRACLE scores.

## Results

### A score balancing immune-stimulatory and immune-suppressive microenvironments outperforms state-of-the-art immune signatures in predicting pan-cancer survival

**Fig. 1:**
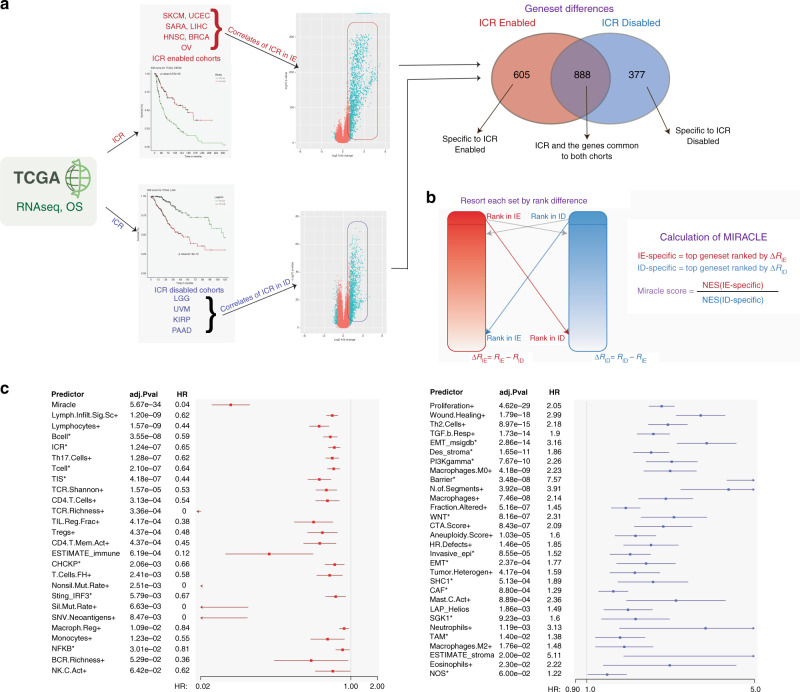
Development of balance score between immune stimulatory and suppressive microenvironments. **a** The rationale and step-by-step workflow of generation of MIRACLE genesets. Venn diagram comparisons show the significant sets in ICR enabled (IE) cohorts (the cohorts where ICR is significantly associated with good prognosis) and ICR disabled (ID) cohorts (the cohorts where ICR is significantly associated with worse prognosis). The genesets are determined by the differential expression between high and low ICR tertiles (log FoldChange >1, *p* value < 0.05 and FDR < 10%). **b** Differentially expressed gene matrices (initially sorted by *p* value) are represented by red and blue rectangles. The genes are then re-ordered using the rank difference in the two lists. The features missed due to hard cut-offs (grey arrows) become lower in rank and are filtered out. IE-specific (red arrows) and ID-specific (blue arrows) genesets are the top genes after Δ*R* reordering. MIRACLE score is the ratio between enrichment scores of the two lists (MIRACLE is short for Mediators of Immune Response Against Cancer in soLid microEnvironments). **c** MIRACLE is compared to a variety of immune-related signatures to predict prognosis in TCGA (*n* = 76 signatures, only significant associations are shown). Hazard ratio for death forest plots represent top performing positive (left) and negative predictors (right) of survival.

Here we characterise samples with a highly active immune phenotype using the ICR.^6,22–24^ The ICR gene signature includes genes involved in T helper type 1 (Th1) and interferon signalling (IFNG, TBX21, CD8A/B, IL12B, STAT1 and IRF1), Th1 chemoattraction (such as the CXCR3 and CCR5 ligands, respectively, CXCL9 and CXCL10, and CCL5) and cytotoxic functions (GNLY, PRF, GZMA, GZMB, and GZMH). ICR signature also includes markers of immuno-suppressive mechanisms such as CD274/PD-L1, PDCD1, CTLA4, FOXP3 and IDO1. We developed a balance score using the approach in Fig. 1a based on ICR. We first identify cancer types from TCGA database in which a highly active immune phenotype is associated with favourable survival (HR < 1 with a *p* value < 0.1; Fig. S1) and cancer types in which this phenotype is associated with decreased survival (HR > 1 with a *p* value < 0.1; Fig. S1). We categorised these two groups of cancer types in ICR-*enabled* (BRCA, SKCM, UCEC, SARC, LIHC, HNSC, OV; abbreviated as “IE”) and ICR-*disabled* (UVM, LGG, PAAD, KIRP; abbreviated as “ID”) groups, respectively as previously described.^24^ All other cancer types in which ICR did not show an association or trend were categorised as ICR-*neutral*. Supervised analysis between samples expressing high levels of the ICR signature versus samples expressing low levels of the ICR signature in enabled tumour types resulted in 1493 differentially expressed genes (DEGs) (log2 fold change ≥1, *p* value ≤ 0.05 and false discovery rate <10%). The same supervised analysis was performed in disabled tumour types obtaining 1265 DEGs associated with high levels of the ICR signature that promote worse prognosis. As expected, the two lists were highly overlapping with 888 common genes, whereas we had 605 genes positively correlated with ICR signature exclusively in the IE cohorts and 377 genes positively correlated with ICR exclusively in ID cohorts (Fig. 1a). We focussed on these last two lists and ranked genes according to their differential expression in the two supervised analyses and scored each gene according to its differential rank in the two lists (see “Methods”). In particular, for each gene of the IE-specific list we compute Δ*R*_IE_ = *R*_IE_ − *R*_ID_ where *R*_IE_ and *R*_ID_ are the ranks of the gene in enabled and disabled differential expression lists, respectively. The top genes re-ranked by Δ*R*_*IE*_ were retained for further analysis. In the same way, the genes in the ID-specific list were scored according to the opposite Δ*R*_ID_  =  *R*_ID_−*R*_IE_. Given a transcriptomics profile, the MIRACLE score is the ratio between the NESs of the whole-gene expression profiles with top genes ordered by Δ*R*_IE_ and Δ*R*_ID_ in IE- and ID-specific gene sets, respectively (Fig. 1b).

MIRACLE score was highly prognostic (HR: 0.043, confidence interval: [0.027, 0.071], *p* value: 6.7 × 10^−36^) in the TCGA cohort (9305 cases, 28 cohorts) (Fig. 1c). The association with survival remained highly significant even if we excluded the 3127 samples used for the identification of the MIRACLE gene signatures (Table S4). When compared with 76 different immune scores recently reported in pan-cancer studies,^5,13^ MIRACLE resulted in the most significant predictor of survival across all the TCGA. We also evaluated MIRACLE at the cohort level, and it was significantly and positively associated with survival in 15 cohorts out of 28 (Fig. S2). Being the largest data set in the ID cohorts, LGG contributes to ID-specific geneset the most and has the highest median value of MIRACLE compared to other cohorts (Fig. S3)

In addition to MIRACLE, 25 other immune-related metrics were significantly found to be associated with better survival (Fig. 1c, Left). However, the significance level of MIRACLE is several orders of magnitude higher than these predictors. On the other hand, 30 other metrics such as “Proliferation”, “Wound Healing”, “Th2 Cells”, “TGFB.Resp” and “Epithelial-to-Mesenchymal Transition” and others were found to be significantly associated with worse survival (Fig. 1c, right).

In order to evaluate whether MIRACLE is an independent survival predictor, we computed likelihood ratio test between different multivariate models including clinical attributes, such as Age, Gender and Stage (see “Methods”). When the survival model, including MIRACLE and cohort as variable (Model 1), was compared to the model including Age, Gender, Stage (Model 2), the clinical attributes did not increase the prognostic value of the model (*p* value: 0.422). On the other hand, MIRACLE significantly improved the significance when we compared Model 2 with the model containing clinical variables only (Model 3) with a *p* value of 1.2 × 10^−35^.

Using PanCancer subtypes, we observed that MIRACLE and survival association is improved in TCGA-BRCA and TCGA-LUAD data sets when the subtypes are included in the survival models. PAM50 classification for Breast Cancer^25^ improved MIRACLE–survival association *p* value from 5.15 × 10^−3^ to 2.04 × 10^−4^ and iCluster classifications for Lung Cancer^26^ improved the survival prediction *p* value from 9.37 × 10^−3^ to 8.54 × 10^−4^. We also observed the effect of PAM50 classification on MIRACLE–survival association in Breast Cancer in METABRIC^27^ and GEO data sets,^28^ in addition to TCGA (data not shown).

We correlated the MIRACLE scores for TCGA cases profiled with both RNAseq and Microrray platforms and found that MIRACLE RNAseq values correlated highly with MIRACLE Microarray values (Affy U133A Platform) (TCGA-LUSC: *n* = 132 patients, *r* = 0.76, *p* value = 1.7 × 10^−26^; TCGA-OV: *n* = 367, *r* = 0.61, *p* value = 2.2 × 10^−39^; TCGA-GBM: *n* = 157, *r* = 0.7, *p* value = 3.2 × 10^−26^), showing independence of MIRACLE score from the gene expression platforms used.

In order to validate MIRACLE’s prognostic value in independent data sets, we collected 55 studies with a total of 7623 patients from GEO. As study selection criteria, we included the microarray platforms with MIRACLE compatible feature IDs (i.e. Affymetrix, Illumina, Entrez IDs or Hugo Gene Symbols as explained in the “Methods” section) but excluded haematological malignancies and cohorts identified as outliers in TCGA analysis (Germ Cell Tumours, Thymomas). The majority (44/55) of the studies used here are also present in the PRECOG database.^14^ In a Cox proportional hazards survival analysis, MIRACLE was strongly associated with better clinical outcome compared to other predictors such as ICR, Estimate^21^ and TIS,^29^ with an HR of 0.1985 and *p* value of 2.73 × 10^−38^ (Fig. S4). Overall, the above results validate and show that MIRACLE outperforms state-of-the-art immune signatures in predicting pan-cancer survival.

### MIRACLE score decouples inflammation from stromal score/TGFβ signalling pathways and other immuno-suppressive signatures

**Fig. 2:**
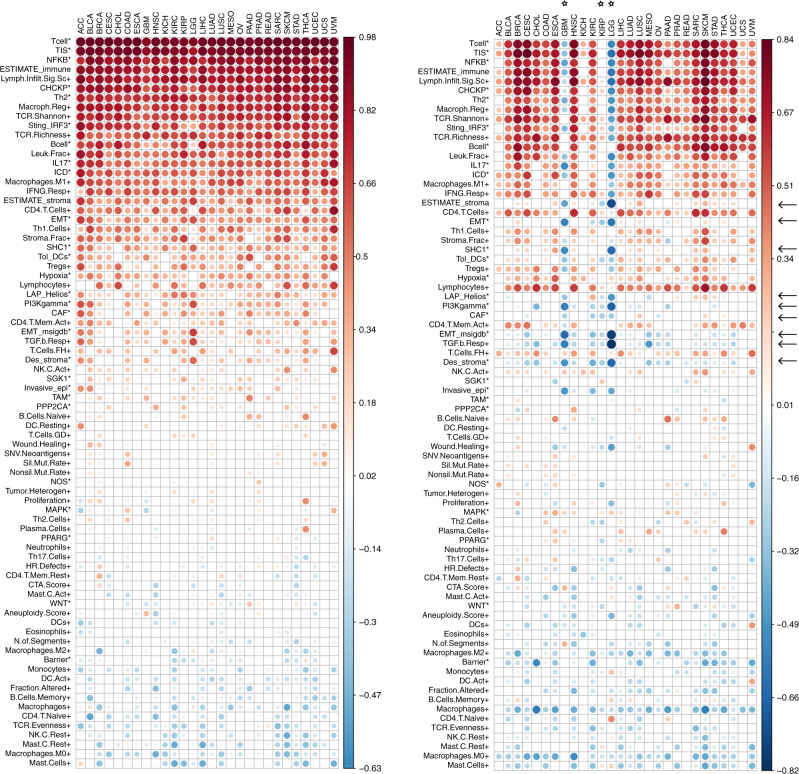
Immune pathways correlate to MIRACLE score. Correlation of ICR enrichment score (left) and MIRACLE score (right) with a set (*n* = 76) of benchmark signatures and metrics (rows) within 28 TCGA cohorts (columns). The colours reflect Pearson correlation coefficients (empty when correlation *p* value is not significant). The rows of both panels are sorted by average correlation coefficients across 28 cohorts of the left (ICR) heatmap. The arrows indicate signatures for which correlation with MIRACLE score deviates from correlation with ICR. Similarly, stars indicate the cohorts where results between MIRACLE and ICR are most divergent.

Since MIRACLE was derived from the DEGs induced by the ICR signature, we analysed the difference between these two scores by evaluating how they correlate with a set of immune-related indices (Fig. 2).^5,13^ As expected, ICR (Fig. 2a) highly correlates with other signatures of T cell infiltration such as Tcell,^30^ TIS,^29^ NFκB^31^ and Checkpoint,^32,33^ as well as ESTIMATE^21^ and Lymph.Infilt.Sig.Sc,^5,13^ across all solid tumours (with a mean correlation coefficient of 0.85 across all mentioned signatures in 28 cohorts). In addition, ICR moderately but consistently correlates with immuno-suppressive signatures such as TGFβ Response,^15^ Cancer-Associated Fibroblasts (CAF),^34,35^ ESTIMATE-Stroma,^21^ Epithelial–Mesenchymal Transition (EMT),^36,37^ PI3Kγ^38–40^ and LAP-Helios (with a mean correlation coefficient of 0.31).^41^ Intriguingly, MIRACLE (Fig. 2b) score is either not correlated or anti-correlated with these inhibitory signatures (mean correlation coefficient: −0.1, Table S5), while it is highly correlated with all the hallmarks of Th1-polarised T cell infiltration (Fig. 2b, Table S5). MIRACLE score correlates with T cell infiltration signatures in all cancer types except for Glioma (LGG and GBM) and Kidney renal papillary cell carcinoma (KIRP). The inverse correlation with Glioma depends on the fact that the immuno-suppressive mechanisms driven by the high presence of myeloid cells,^42,43^ especially in the IDH wild-type tumours having a worse survival,^44^ tend to enrich the denominator of the MIRACLE. Similarly, a large proportion (about 42%) of KIRP tumours is characterised by the presence of high immune infiltration concomitant with a suppressive microenvinment.^45^

**Fig. 3:**
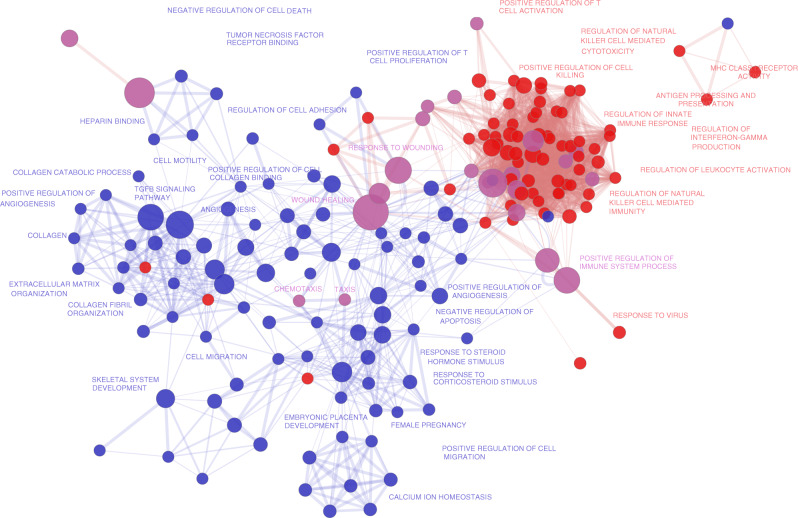
Functional enrichments of MIRACLE signatures. Network visualisation of gene ontology (GO) terms enriched in IE-specific (red nodes/edges/text) or ID-specific gene lists (blue nodes/edges/text). Each node represents an enriched GO term and its size correlates with geneset size and the edges represent the relatedness (overlap) of the GO terms. IE- and ID-specific GO terms cluster separately in a self-organising fashion. Purple nodes are the GO terms enriched in both IE- and ID-specific genesets. Only selected nodes are labelled due to space limit.

To better clarify the functional role of the signatures that define the MIRACLE score, we performed enrichment analysis of GO categories of the list of genes specific of ICR-Enabled tumour types (605 genes) and the list specific of ICR-Disabled list (377 genes) (Fig. 3). We found that the ICR-Enabled list was associated with “T cell activation”, “Regulation of Lymphocyte Mediated Immunity” and “Adaptive Immune Response”, as well as “Positive Regulation of Cell Killing”, among others. On the other hand, the ICR-Disabled list was associated with GO terms including “Negative Regulation of Cell Death”, “TGFB Signalling Pathway” and “Epithelial-to-Mesenchymal Transition” (EMT), which are involved in tumour progression with metastatic expansion, and the generation of tumour cells with stem cell properties that play a major role in resistance to cancer treatment.^46,47^ In the self-organising network plot of these GO terms, IE and ID signatures reflect two opposing networks, clustering separately (Fig. 3). Interestingly the GO terms enriched in both IE and ID lists include “Chemotaxis” and “Taxis”, which are functions that result in the presence of immune infiltrate (hence high ICR in both IE and ID cohorts) but do not reflect activatory or suppressive mediators of immune response against cancer. These results suggest that, within a given sample, MIRACLE score measures the existing balance between two opposing immune-related phenotypes.

### Genomics events associated with MIRACLE score

**Fig. 4:**
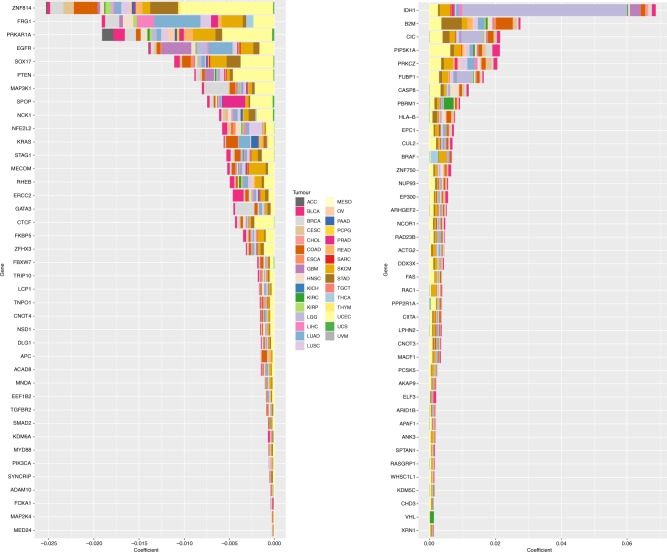
Genomic events positively and negatively associated with MIRACLE. Top 40 of the mutated genes with negative non-zero coefficients of a trained elastic net model. One hundred and eighty-six identified genes whose mutation is associated with a decrease of the MIRACLE (left panel). Top 40 mutated genes with a positive association with MIRACLE score in pan-cancer trained model (right panel). Contributions of each individual cancer type to the coefficient in trained elastic net model are proportionally indicated by the size of the bars.

We then explored the association of specific oncogenic mutations with MIRACLE score. We first selected a set of 470 frequently mutated genes in cancer,^48^ then trained an elastic net model to predict our score as a function of mutations in each sample and using the cohort as covariate.^49^ The positive coefficients of the trained model were used to identify genes whose mutations are associated with an increase of the MIRACLE score and negative coefficients identify the genes whose mutations are associated with a decrease of the MIRACLE score (Fig. 4). We evaluated the accuracy of the model in a tenfold cross validation computing the correlation between the model prediction and the true MIRACLE scores obtaining a Spearman correlation of 0.78 ± 0.017 (*p* value ~0). Genes associated with a decrease of MIRACLE score include: EGFR, PRKAR1A, ZNF814, PTEN, MAP3K1, CHEK2, FRG1, KRAS, MAP4K1, NF1, PIK3CA, and others. Interestingly, several of these genes, such as EGFR,^50^ have previously been associated with tumour-intrinsic factors modulating cancer immunity activating immune-suppressive phenotype. For example, it has been recently shown that the mutations in PRKAR1A are a tumour intrinsic event leading to drastic alterations in the genetic programme of cancer cells, thereby remodelling the tumour microenvironment.^51^ MAP3K1 mutations have an effect on low Th1 polarisation in breast cancer.^6^

The top genes of which mutations positively correlate with MIRACLE include genes that are associated with differential survival in cancer subtypes, such as IDH1, CIC and FUBP1 in glioma,^44^ whereas other genes associated with immune-evasion mechanisms that follow immunologic pressure such as mutations of antigen-presenting machinery transcripts previously described (i.e., B2M, VHL and CASP8).^52^

### Predictive value of MIRACLE score in immune checkpoint therapy

**Fig. 5:**
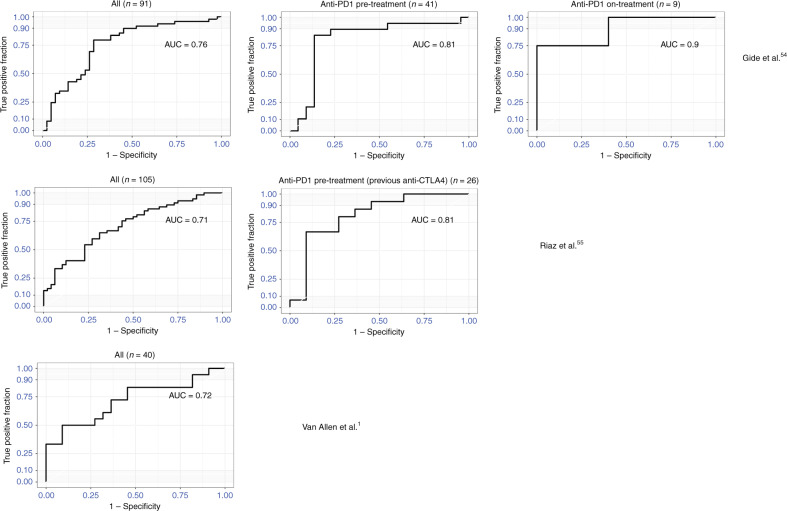
MIRACLE predicts ICI response. ROC curves showing predictive value of MIRACLE in four melanoma immunotherapy response data sets. Responders are defined as complete response, partial response, or stable disease, and non-responders are defined as patients with progressive disease. Top: Gide et al., all samples (left), anti-PD1 pre-treatment (middle) and anti-PD1 on-treatment samples (right).^53^ Middle: Riaz et al. all samples (left) and samples from patients previously treated with anti-CTLA4.^54^ Bottom: Van Allen et al., data set. The patient counts are indicated for each cohort in parentheses (*n*).

In the previous sections, we have shown the prognostic impact of MIRACLE and its associated biology. To define the clinical relevance of the MIRACLE score in the setting of immune checkpoint treatment, we evaluated the predictive value of MIRACLE score across multiple public data sets of anti-CTLA4 and anti-programmed cell death protein 1 (anti-PD1) treatment. Gide et al.^53^ profiled 91 samples (pre-treatment or on-treatment) from anti-PD1 or anti-PD1/anti-CTLA4 combo-treated melanoma patients. MIRACLE scores are significantly higher in responders across all samples and when pre-treatment and on-treatment samples were analysed separately (Fig. S5A, B). AUC of prediction is 0.78 for all patients, while in the anti-PD1-treated subset, it reaches 0.81 (0.81 for pre-treatment and 0.9 on-treatment) (Fig. 5a). Interestingly, the MIRACLE score is not predictive of response in anti-PD1/anti-CTLA4 combo-treated patients using either pre-treatment or on-treatment samples. One possibility is that the combination of anti-PD1 and anti-CTLA4 overcomes the immunosuppression that MIRACLE accounts for, which might be the cause for the lack of association in this subset. Riaz et al.^54^ profiled 109 pre-treatment or on-treatment samples from anti-PD1-treated melanoma patients. Again, MIRACLE scores are significantly higher in responders, across all samples and in pre-treatment and on-treatment subsets (*p* values 6.14 × 10^−5^, 0.022 and 0.001, respectively; Fig. S5B; the AUC of values of the MIRACLE prediction is 0.71, 0.73 and 0.68, respectively). When the samples are subset based on previous checkpoint therapy (anti-CTLA4), MIRACLE is selectively associated with response in checkpoint therapy progressed samples (*p* value: 2.22 × 10^−4^, AUC: 0.81; Figs. 5b and S5A). MIRACLE has no significant predictive value in the relatively smaller Hugo et al. data set (Fig. S5A).^55^ On the contrary, another method that quantifies T cell dysfunction, TIDE, is predictive of response in the Hugo et al. data set. Additionally, TIDE only has predictive value in previous checkpoint therapy-naive cases of Riaz et al. data set but no predictive value in patients who progressed after immunotherapy.^55,56^ This suggests that MIRACLE and TIDE methods can potentially complement each other. Similarly, Dizier et al.^57^ profiled 65 pre-treatment melanoma samples from patients who were treated with recombinant MAGE-A3 antigen immunotherapy using microarrays. MIRACLE scores were significantly higher in responders (*t* test *p* value: 0.0022) with an AUC of 0.77 (Fig. S5A, B). Another gene expression data set of 42 melanoma patients treated with anti-CTLA4 was reported in Van Allen et al.^1^ Our scores are significantly associated with response (*p* value: 0.009) as well as overall survival (*p* value: 0.013 Cox proportional hazard) and AUC 0.72 (Fig. 5c). In addition, we tested MIRACLE in a non-small cell lung cancer data set of 11 (*n*: 16 when including FFPE samples) pre-treatment samples from anti-PD10-treated patients, where MIRACLE was associated with response (*p* value: 0.042, ref. ^58^). Overall, considering the various stratifications of the publicly available response data sets, MIRACLE, ICR and TIS were predictive with the most number of data sets when compared to other immune-related signatures (Fig. S5).

## Discussion

Here we have shown that a balance score encapsulating both immune-active and immune-repressive cancer phenotype defines a continuum of clinically and biologically relevant classes of tumours. We derived our score by evaluating the differences between the transcriptional programmes associated with the presence of a pro-survival Th1-polarised immune infiltration and the transcriptional programmes activated in tumour types where the immune microenvironment has a negative effect on prognosis. Our score displayed the highest positive association with survival when compared with several immune phenotyping scores reported recently in large scale studies.^13^ and was significantly associated with better survival in 15 out 28 TCGA indications; its prognostic features were confirmed in 55 data sets spanning 11 tumour types and 7623 cases. It decouples the typical immune-regulatory mechanisms that often are simultaneously active in T cell-inflamed microenvironments. This explains why it always results in a positive HR. Indeed, immune homoeostasis is a subtle balance between the immune defence against foreign pathogens and suppression of the immune system to maintain self-tolerance and prevent autoimmune disease.^59^

We interrogated the association between the tumour composition and the increase or decrease of our score. Interestingly, by using an elastic net regression approach we found known and novel alterations consistently associated with a desert immune phenotype. The top genes whose mutations are associated with a decrease of MIRACLE were EGFR, PRKAR1A, ZNF814, PTEN, TRIP10, MECOM, MAP3K1, MYD88, CNOT4 and NCK1. The association of EGFR with an immune-desert tumour phenotype with poor response to checkpoint inhibitor therapies has been observed in several clinical trials.^60,61^ Mutations of PRKAR1A have been recently demonstrated to drive a drastic remodelling of the tumour microenvironment. Loss of PTEN expression has been shown to prime the microenvironment for tumour development through several mechanisms, such as modification of the pattern of cytokine secretion creating an immune-suppressive microenvironment with increase of immune cell populations that can promote tumour progression.^62^ Enrichment of PTEN mutations driving an immuno-suppressive microenvironment with higher expression of M2 macrophages have been observed in non-responder to checkpoint inhibitor therapies on glioma.^63^ Mutations of MAP3K1 are associated with immune-desert phenotype in breast.^6^ The reported association could represent novel tumour-intrinsic mechanisms deserving further experimental studies.

We have also reported that MIRACLE score is a predictive biomarker of response to ICIs in public data sets. Several transcriptional-based signatures have been recently proposed and validated in clinical trials^64^ or retrospectively.^16,17^ One of the main differences between the proposed score and other similar approaches^16,17^ is that MIRACLE score does not need any data set-dependent normalisation and can be independently and incrementally applied patient by patient as in a typical clinical trial scenario to stratify patients who will be more likely to benefit from PD-1 or CTLA-4 checkpoint immunotherapies. Several limitations and possible extensions of the analysis reported in this paper should also be mentioned. First, our study was primarily focussed on gene expression biomarkers. Other data types such as protein levels and somatic mutations could eventually improve the ability of our method to predict ICI response, and therefore one of the possible extensions of the method to multi-omics variables will be considered in the follow-up. Another limitation that could eventually lead to further improvements is that the signatures that define the MIRACLE score were selected just considering the effect of immune system polarisation on survival. As more and more response data sets are being made available from clinical trials, these data could be retrospectively used for refining the immune signatures and designing better response predictors.

To enable testing of MIRACLE by clinicians and the public, we created a web application for response prediction using transcriptome profiles at https://miracle.shinyapps.io/miracle_shinyapp/. MIRACLE can potentially help physicians to predict survival and ICI response for defining patient selection strategies.

In conclusion, MIRACLE features have been derived in a genome-wide and pan-cancer manner and not been limited to any prior knowledge of gene features or cancer types other than measurable immune phenotypes and patient outcomes. Thus MIRACLE can be applied to any bulk RNA measurement to stratify patients to predict outcomes and checkpoint inhibitor therapy responses.

## Supplementary information

Supplementary Figures and Tables

## Data Availability

Raw and source data availability: all raw data used in the study (weblinks, references, cohort sizes and other meta-data attributes) are listed in Table S1.

## Code availability

The source code and installation instructions for MIRACLE R package can be found publicly at GitHub (https://github.com/tolgaturan-github/Miracle). MIRACLE score can be calculated for a normalised RNAseq or Microarray gene expression matrix allowing the use of various platform identifiers including Ensembl Gene Ids (preferred), U133p2 and U133A from Affymetrix, IlluminaHTv3 and IlluminaHTv4 from Illumina and other expression data sets with features mapped to Entrez IDs or Hugo Gene Symbols. A shinyapp is also available at the following address https://miracle.shinyapps.io/miracle_shinyapp/, to enable the use of MIRACLE by non-bioinformaticians.
