# Correction: A balance score between immune stimulatory and suppressive microenvironments identifies mediators of tumour immunity and predicts pan-cancer survival

**DOI:** 10.1038/s41416-020-01193-w

**Published:** 2020-12-04

**Authors:** Tolga Turan, Sarah Kongpachith, Kyle Halliwill, Jessica Roelands, Wouter Hendrickx, Francesco M. Marincola, Thomas J. Hudson, Howard J. Jacob, Davide Bedognetti, Josue Samayoa, Michele Ceccarelli

**Affiliations:** 1 Computational Immunology and Oncology (CIAO), AbbVie, Redwood City, CA USA; 2 Cancer Research Department, Sidra Medicine, Doha, Qatar; 3 Department of Surgery, Leiden University Medical Center (LUMC), Leiden, The Netherlands; 4 Refuge Biotechnologies, Menlo Park, CA USA; 5 Genomics Research Center (GRC), AbbVie, Lake County, IL USA; 6 Dipartimento di Medicina Interna e Specialità Mediche, Università degli Studi di Genova, Genova, Italy; 7 Department of Electrical Engineering and Information Technology, University of Naples “Federico II”, Naples, Italy; 8 Istituto di Ricerche Genetiche “G. Salvatore”, Biogem s.c.ar.l, 83031 Ariano Irpino, Italy

**Keywords:** Classification and taxonomy, Cancer genomics, Immunotherapy, Bioinformatics

Correction to: *British Journal of Cancer* (2020); 10.1038/s41416-020-01145-4, published online 5 November 2020

Since the publication of this paper the authors have noticed an error in the name of Wouter Hendrickx, where Dr. Hendrickx’s name was incorrectly listed as Wouter Hendricks. This has been corrected above.

