# Supplementary material for: A balance score between immune stimulatory and suppressive microenvironments identifies mediators of tumour immunity and predicts pan-cancer survival
Source: Br J Cancer. 2020 Nov 5;124(4):760–9. doi: 10.1038/s41416-020-01145-4 (PMC7884411; doi:10.1038/s41416-020-01145-4)
Supplement: Supplementary file 1 — Supplementary Figures and Tables [file 41416_2020_1145_MOESM1_ESM.docx]

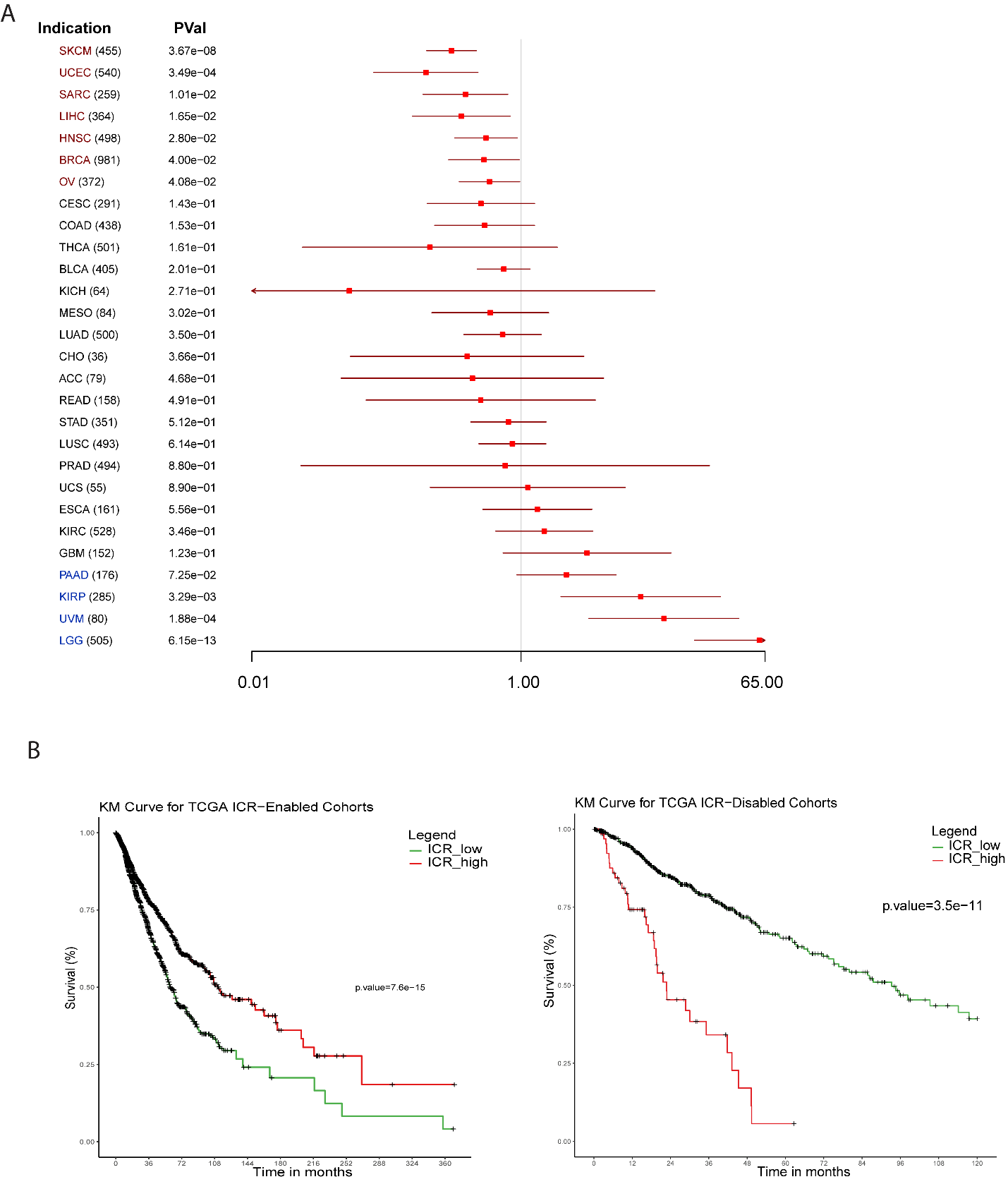


**Figure S1. ICR Enabled and Disabled Tumors**. Forest plot of Hazard Ratios showing association of ICR and survival in 28 TCGA Cohorts (A). In 7 TCGA Cohorts, ICR is significantly associated with good prognosis (**ICR Enabled** cohorts). Conversely, in 4 Cohorts ICR is significantly associated with worse prognosis (**ICR Disabled** cohorts). The patient counts are indicated for each cohort in parentheses (n). B: Kaplan-Meier curves for high ICR and low ICR patients in IE and ID cohorts.


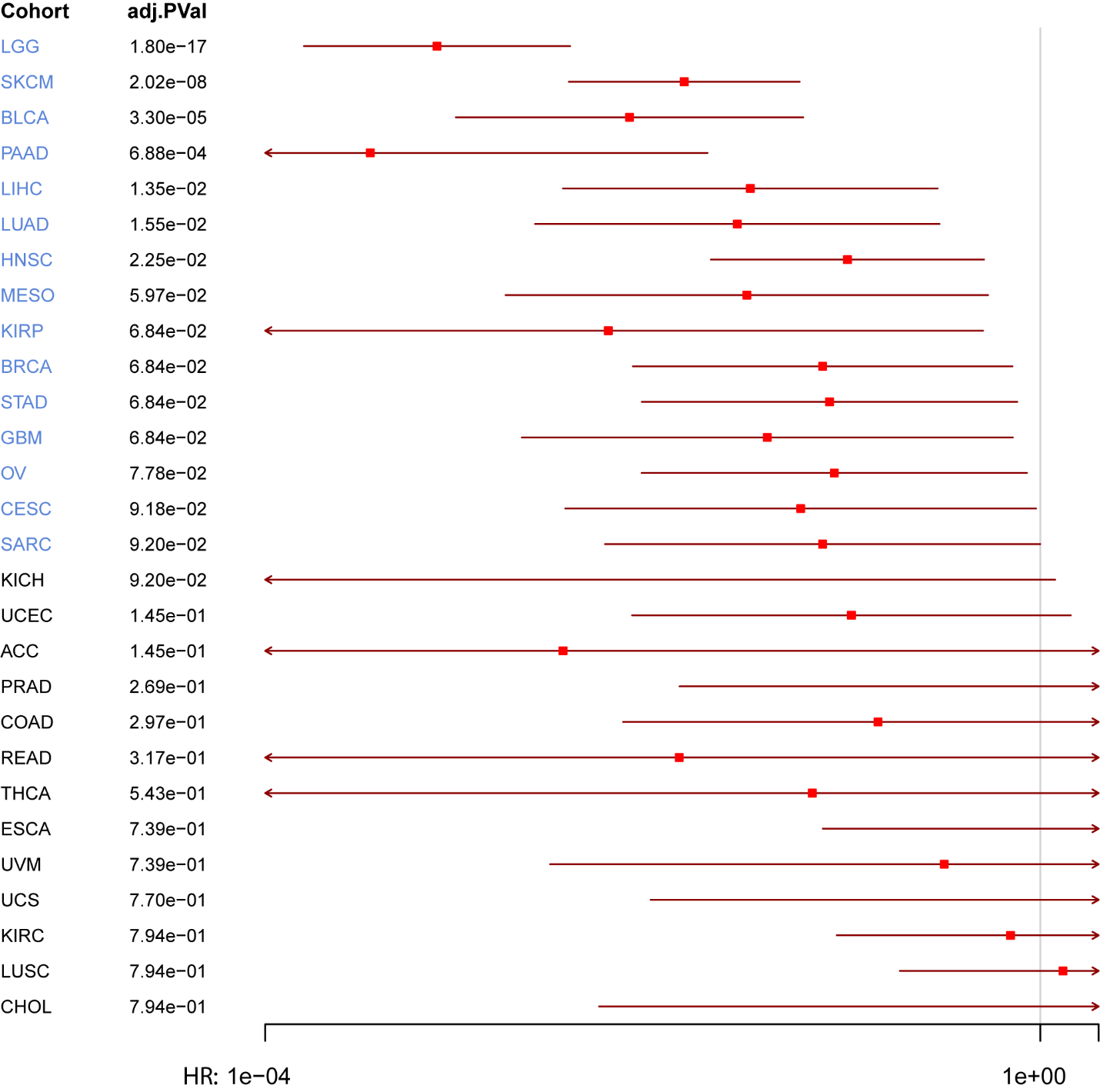


**Figure S2. MIRACLE prognosis per cancer**. Forest plot of Hazard Ratios showing association of Miracle and survival in 28 TCGA Cohorts. Miracle is significantly and invariably associated with good prognosis in 15 TCGA Cohorts.

**
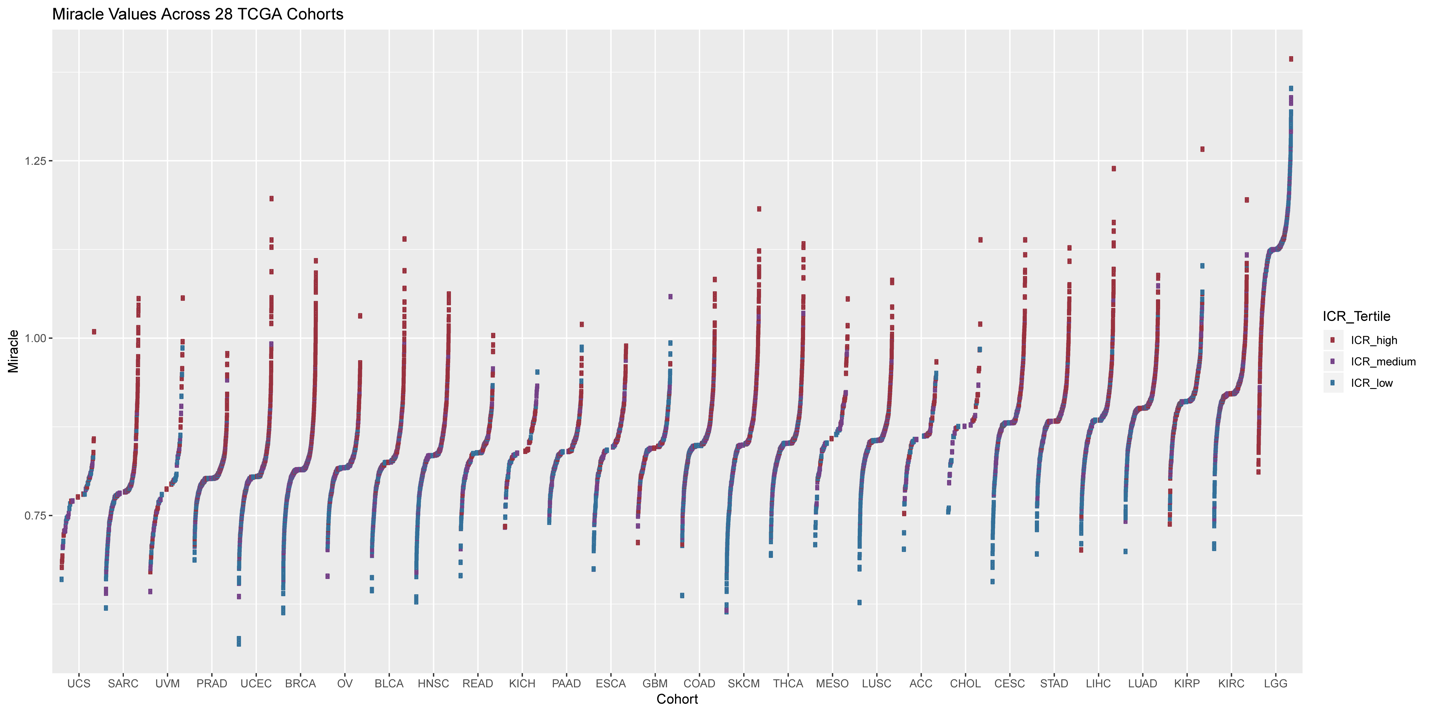
**

**Figure S3. Distribution of MIRACLE scores and corresponding ICR groups (tertiles).** MIRACLE values from each cohort are sorted and colored by ICR tertile.


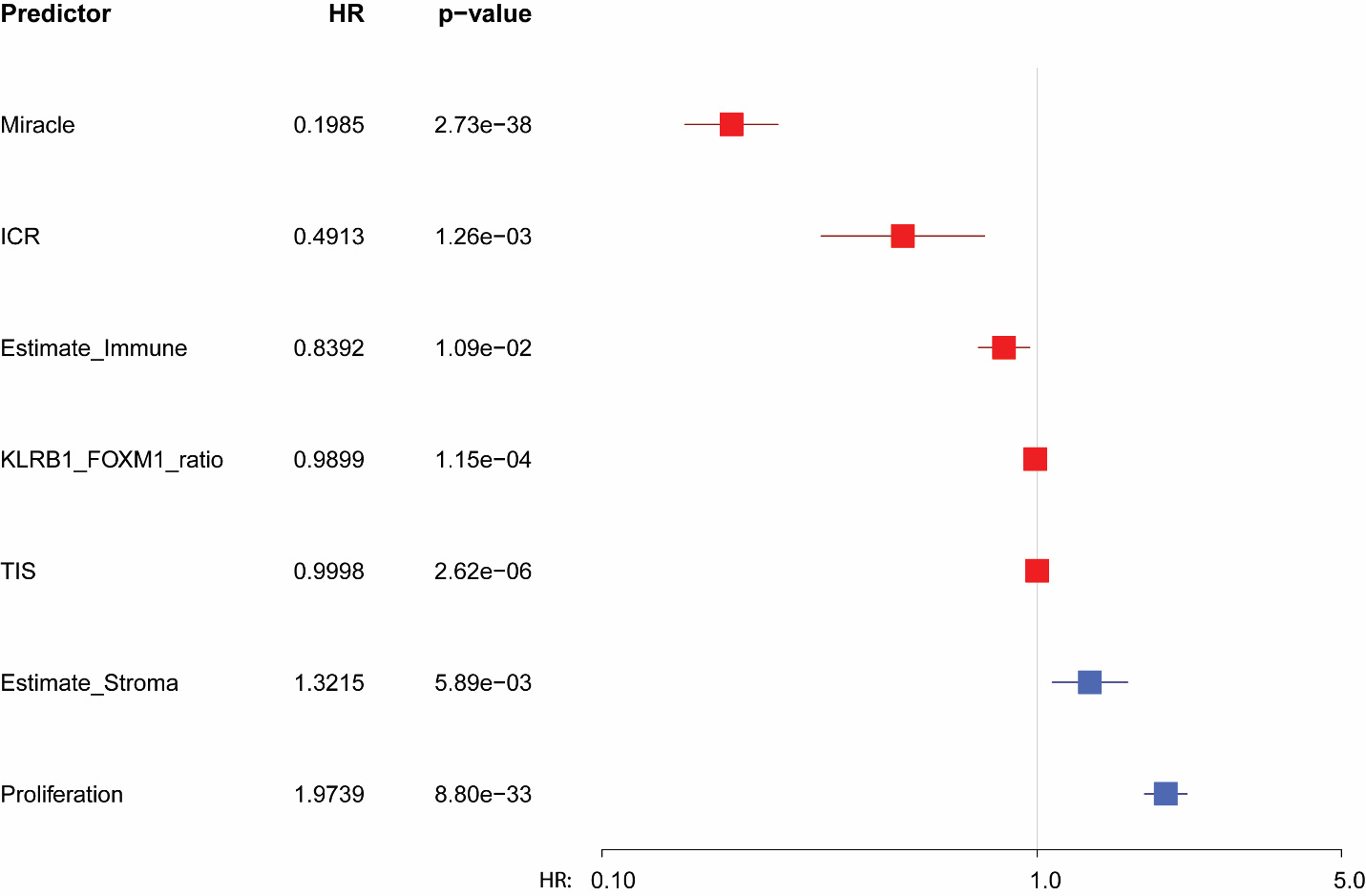


**Figure S4. Prognostic prediction in validation datasets.** Forest plot of Hazard Ratios showing association of Miracle and survival in validation datasets.

A
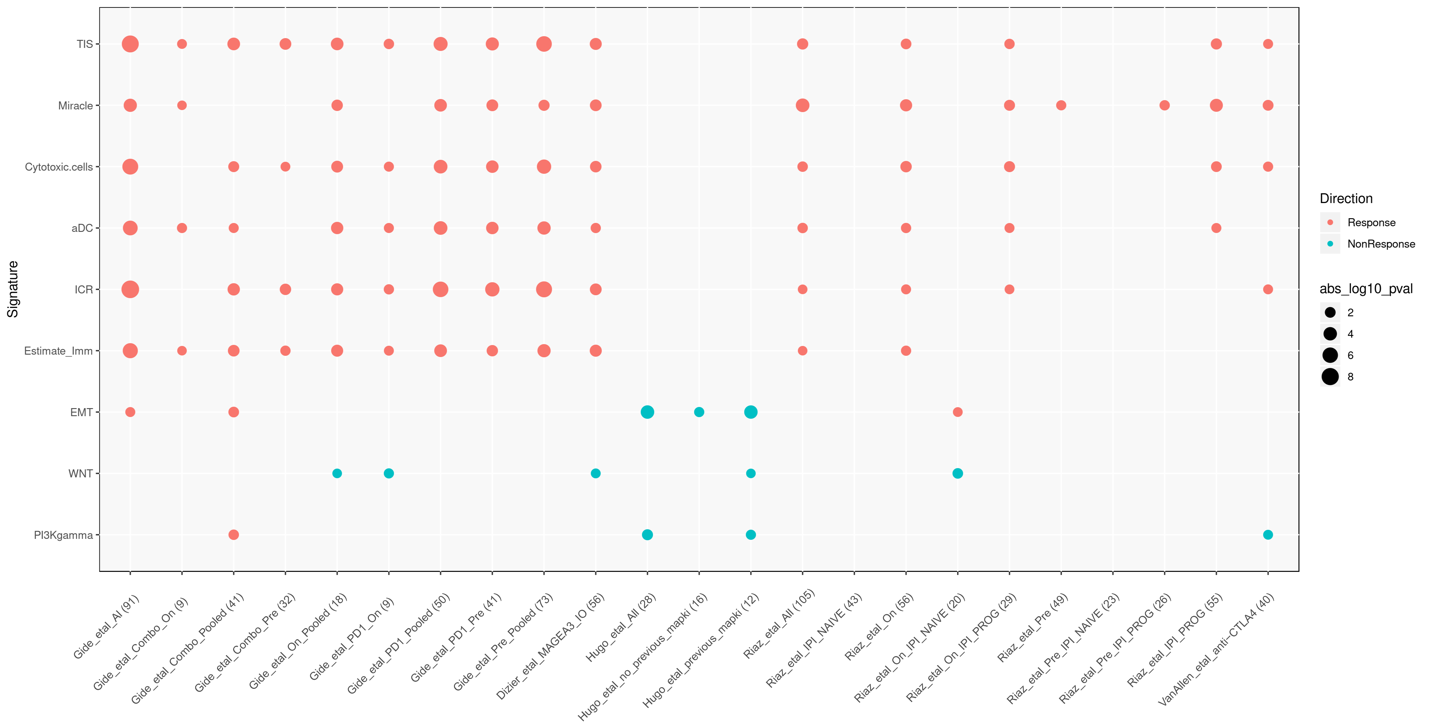


B
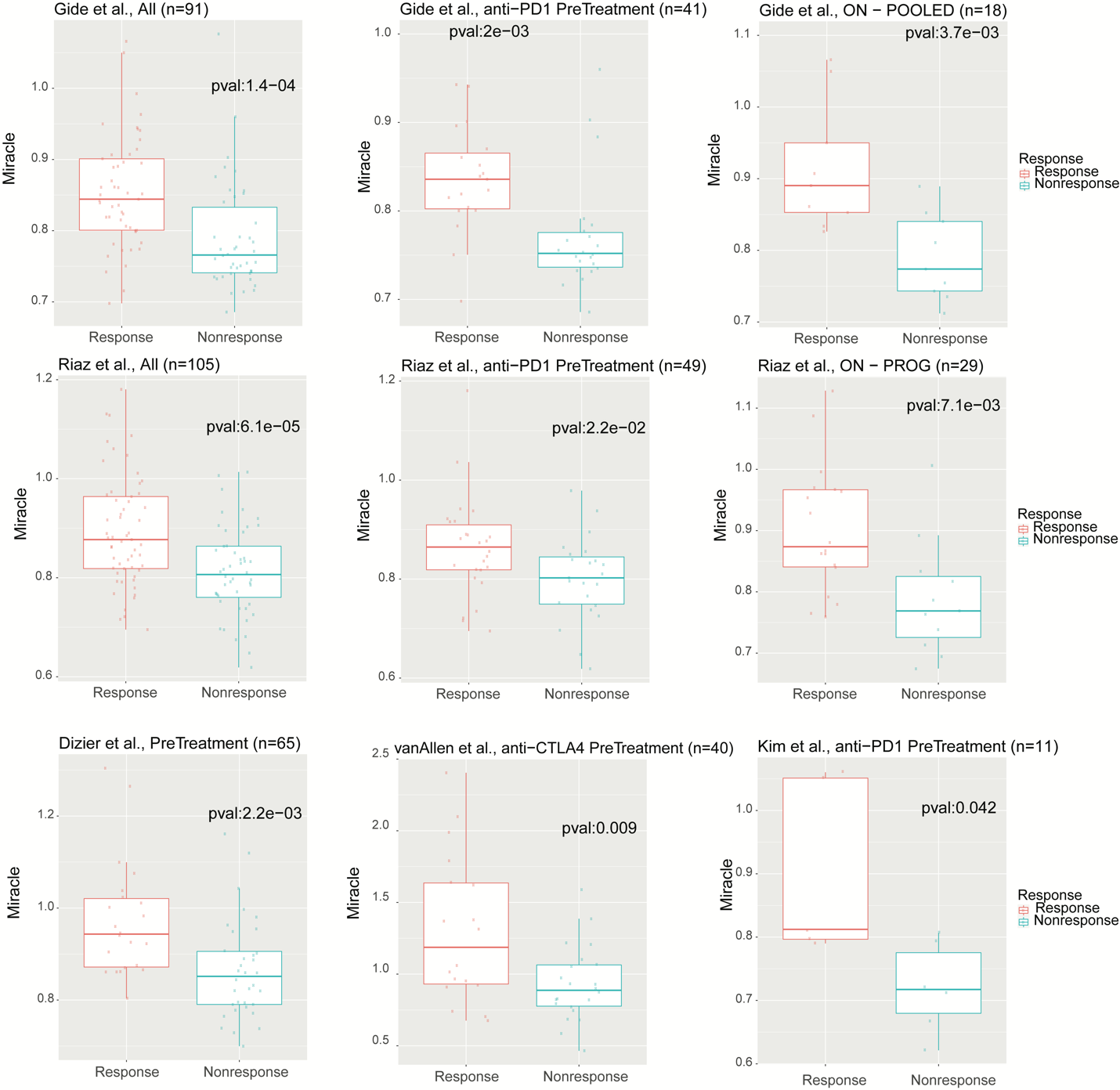


**Figure S5. MIRACLE association with response in cohorts from immunotherapy treated patients. A:** Miracle scores in 5 melanoma and 1 lung cancer immunotherapy response datasets and the association with ICI response in various stratifications (total of 24 subsets). **B:** Boxplot representation of MIRACLE in main response datasets shown in A. The patient counts are indicated for each cohort in parentheses (n).

**Supplementary Tables:**

**Table S1**. Public Datasets Used. Descriptive information and links to original sources for the public datasets used in the study.

| **DataSet ID** | **Authors** | **Input Data** | **Cohort** | **Count** | **Outcome** | **Link** |
| --- | --- | --- | --- | --- | --- | --- |
| TCGA | Various | Raw Integer Counts | Various | 10079 | Overall Survival | https://gdc.cancer.gov/ |
| GSE91061 | Riaz et al.,2017 | Raw .fastq Files | Melanoma | 109 | anti-PD1 Response | https://www.ncbi.nlm.nih.gov/geo/query/acc.cgi?acc=GSE91061 |
| PRJEB23709 | Gide et al.,2019 | Raw .fastq Files | Melanoma | 91 | anti-PD1 or anti-PD1/CTLA4 Response | https://www.ebi.ac.uk/ena/data/view/ERX2263292 |
| GSE78220 | Hugo et al., 2016 | Raw .fastq Files | Melanoma | 28 | anti-PD1 Response | https://www.ncbi.nlm.nih.gov/geo/query/acc.cgi?acc=GSE78220 |
| GSE35640 | Ulloa-Montoya et al.,  2013 | Raw .CEL Files | Melanoma | 65 | MAGE-A3 Immunotherapy Response | https://www.ncbi.nlm.nih.gov/geo/query/acc.cgi?acc=GSE35640 |
| phs000452.v2.p1 | vanAllen et al., 2015 | Normalized Expression Matrix | Melanoma | 42 | anti-CTLA4 Response and Survival | http://tide.dfci.harvard.edu/login/ |
| GSE126044 | Kim et al., 2020 | Raw Integer Counts | Lung Cancer | 16 | anti-PD1 Response | <https://www.ncbi.nlm.nih.gov/geo/query/acc.cgi?acc=GSE126044> |
| GSE108474 | Gusev et al., 2018 | Raw .CEL Files | Glioma | 490 | Overall Survival | https://www.ncbi.nlm.nih.gov/geo/query/acc.cgi?acc=GSE108474 |
| GSE84437 | Cheung et al., not yet pub | Normalized Expression Matrix | Gastric Cancer | 433 | Overall Survival | https://www.ncbi.nlm.nih.gov/geo/query/acc.cgi?acc=GSE84437 |
| GSE32894 | Sjödahl et L., 2012 | Normalized Expression Matrix | Bladder Cancer | 224 | Overall Survival | https://www.ncbi.nlm.nih.gov/geo/query/acc.cgi?acc=GSE32894 |
| GSE14520 | Roessler et al., 2012 | Raw .CEL Files | Hepatocelluler Cancer | 242 | Overall Survival | https://www.ncbi.nlm.nih.gov/geo/query/acc.cgi?acc=GSE14520 |
| GSE17538 | Smith et al., 2010 | Raw .CEL Files | Colon Adenocarcinoma | 232 | Overall Survival | https://www.ncbi.nlm.nih.gov/geo/query/acc.cgi?acc=GSE17538 |
| GSE65904 | Cirenajwis et al., 2015 | Normalized Expression Matrix | Melanoma | 210 | Overall Survival | https://www.ncbi.nlm.nih.gov/geo/query/acc.cgi?acc=GSE65904 |
| GSE26712 | Bonome et al., 2008 | Raw .CEL Files | Ovarian Cancer | 185 | Overall Survival | https://www.ncbi.nlm.nih.gov/geo/query/acc.cgi?acc=GSE26712 |
| GSE31210 | Yamauchi et al., 2012 | Raw .CEL Files | Lung Adenocarcinoma | 226 | Overall Survival | https://www.ncbi.nlm.nih.gov/geo/query/acc.cgi?acc=GSE31210 |
| GSE39582 | Marisa et al., 2013 | Normalized Expression Matrix | Colon Adenocarcinoma | 579 | Overall Survival | <https://www.ncbi.nlm.nih.gov/geo/query/acc.cgi?acc=GSE39582> |
| GSE72094 | Schabath et al., 2016 | Normalized Expression Matrix | Lung Adenocarcinoma | 398 | Overall Survival | <https://www.ncbi.nlm.nih.gov/geo/query/acc.cgi?acc=GSE72094> |
| GSE9891 | Tothill et al., 2008 | Normalized Expression Matrix | Ovarian Cancer | 278 | Overall Survival | <https://www.ncbi.nlm.nih.gov/geo/query/acc.cgi?acc=GSE9891> |
| GSE30219 | Rousseaux et al., 2013 | Normalized Expression Matrix | Lung Cancer | 293 | Overall Survival | <https://www.ncbi.nlm.nih.gov/geo/query/acc.cgi?acc=GSE30219> |
| GSE65858 | Wichmann et al., 2015 | Normalized Expression Matrix | Head and Neck Cancer | 270 | Overall Survival | <https://www.ncbi.nlm.nih.gov/geo/query/acc.cgi?acc=GSE65858> |
| GSE13507 | Kim et al., 2010 | Normalized Expression Matrix | Bladder Cancer | 165 | Overall Survival | <https://www.ncbi.nlm.nih.gov/geo/query/acc.cgi?acc=GSE13507> |
| GSE3494 | Miller et al., 2005 | Normalized Expression Matrix | Breast Cancer | 251 | Overall Survival | <https://www.ncbi.nlm.nih.gov/geo/query/acc.cgi?acc=GSE3494> |
| GSE7390 | Desmedt et al., 2007 | Normalized Expression Matrix | Breast Cancer | 198 | Overall Survival | <https://www.ncbi.nlm.nih.gov/geo/query/acc.cgi?acc=GSE7390> |
| GSE13041 | Lee et al., 2009 | Normalized Expression Matrix | Glioma | 218 | Overall Survival | <https://www.ncbi.nlm.nih.gov/geo/query/acc.cgi?acc=GSE13041> |
| GSE24450 | Heikkinen et al., 2011 | Normalized Expression Matrix | Breast Cancer | 183 | Overall Survival | <https://www.ncbi.nlm.nih.gov/geo/query/acc.cgi?acc=GSE24450> |
| GSE1456 | Pawitan et al., 2005 | Normalized Expression Matrix | Breast Cancer | 159 | Overall Survival | <https://www.ncbi.nlm.nih.gov/geo/query/acc.cgi?acc=GSE1456> |
| GSE19188 | Hou et al., 2010 | Normalized Expression Matrix | Lung Cancer | 82 | Overall Survival | <https://www.ncbi.nlm.nih.gov/geo/query/acc.cgi?acc=GSE19188> |
| GSE3149 | Bild et al., 2006 | Normalized Expression Matrix | Ovarian Cancer | 141 | Overall Survival | <https://www.ncbi.nlm.nih.gov/geo/query/acc.cgi?acc=GSE3149> |
| GSE8894 | Lee et al., 2008 | Normalized Expression Matrix | Lung Cancer | 138 | Overall Survival | <https://www.ncbi.nlm.nih.gov/geo/query/acc.cgi?acc=GSE8894> |
| GSE14814 | Zhu et al., 2010 | Normalized Expression Matrix | Lung Cancer | 90 | Overall Survival | <https://www.ncbi.nlm.nih.gov/geo/query/acc.cgi?acc=gse14814> |
| GSE4573 | Raponi et al., 2006 | Normalized Expression Matrix | Lung Cancer | 130 | Overall Survival | <https://www.ncbi.nlm.nih.gov/geo/query/acc.cgi?acc=GSE4573> |
| GSE16446 | Li et al., 2010 | Normalized Expression Matrix | Breast Cancer | 107 | Overall Survival | <https://www.ncbi.nlm.nih.gov/geo/query/acc.cgi?acc=GSE16446> |
| GSE3141 | Bild et al., 2006 | Normalized Expression Matrix | Lung Cancer | 111 | Overall Survival | <https://www.ncbi.nlm.nih.gov/geo/query/acc.cgi?acc=GSE3141> |
| GSE4271 | Phillips et al., 2006 | Normalized Expression Matrix | Glioma | 77 | Overall Survival | <https://www.ncbi.nlm.nih.gov/geo/query/acc.cgi?acc=GSE4271> |
| GSE20486 | Parris et al., 2010 | Normalized Expression Matrix | Breast Cancer | 97 | Overall Survival | <https://www.ncbi.nlm.nih.gov/geo/query/acc.cgi?acc=GSE20486> |
| GSE31056 | Reis et al., 2011 | Normalized Expression Matrix | Head and Neck cancer | 96 | Overall Survival | <https://www.ncbi.nlm.nih.gov/geo/query/acc.cgi?acc=GSE31056> |
| GSE24550 | Agesen et al., 2012 | Normalized Expression Matrix | Colon Adenocarcinoma | 77 | Overall Survival | <https://www.ncbi.nlm.nih.gov/geo/query/acc.cgi?acc=GSE24550> |
| GSE4412 | Freije et al., 2004 | Normalized Expression Matrix | Glioma | 85 | Overall Survival | <https://www.ncbi.nlm.nih.gov/geo/query/acc.cgi?acc=GSE4412> |
| GSE7696 | Murat et al., 2008 | Normalized Expression Matrix | Glioma | 80 | Overall Survival | <https://www.ncbi.nlm.nih.gov/geo/query/acc.cgi?acc=GSE8401> |
| GSE8401 | Xu et al., 2008 | Normalized Expression Matrix | Melanoma | 67 | Overall Survival | <https://www.ncbi.nlm.nih.gov/geo/query/acc.cgi?acc=GSE8401> |
| GSE24549 | Agesen et al., 2012 | Normalized Expression Matrix | Colon Adenocarcinoma | 83 | Overall Survival | <https://www.ncbi.nlm.nih.gov/geo/query/acc.cgi?acc=GSE24549> |
| GSE14764 | Denkert et al., 2009 | Normalized Expression Matrix | Ovarian Cancer | 80 | Overall Survival | <https://www.ncbi.nlm.nih.gov/geo/query/acc.cgi?acc=GSE14764> |
| GSE18521 | Mok et al., 2009 | Normalized Expression Matrix | Ovarian Cancer | 53 | Overall Survival | <https://www.ncbi.nlm.nih.gov/geo/query/acc.cgi?acc=GSE18521> |
| GSE16581 | Lee et al., 2010 | Normalized Expression Matrix | Meningioma | 67 | Overall Survival | <https://www.ncbi.nlm.nih.gov/geo/query/acc.cgi?acc=GSE16581> |
| GSE1993 | Petalidis et al., 2008 | Normalized Expression Matrix | Astrocytoma | 60 | Overall Survival | <https://www.ncbi.nlm.nih.gov/geo/query/acc.cgi?acc=GSE1993> |
| GSE10927 | Giordano et al., 2009 | Normalized Expression Matrix | Adrenocortical Carcinoma | 24 | Overall Survival | <https://www.ncbi.nlm.nih.gov/geo/query/acc.cgi?acc=GSE10927> |
| GSE12945 | Staub et al., 2009 | Normalized Expression Matrix | Colon Adenocarcinoma | 62 | Overall Survival | <https://www.ncbi.nlm.nih.gov/geo/query/acc.cgi?acc=GSE12945> |
| GSE10245 | Kuner et al., 2009 | Normalized Expression Matrix | Lung Cancer | 58 | Overall Survival | <https://www.ncbi.nlm.nih.gov/geo/query/acc.cgi?acc=GSE10245> |
| GSE22155 | Jönsson et al., 2010 | Normalized Expression Matrix | Colon Adenocarcinoma | 76 | Overall Survival | <https://www.ncbi.nlm.nih.gov/geo/query/acc.cgi?acc=GSE22155> |
| GSE29013 | Xie et al., 2011 | Normalized Expression Matrix | Lung Cancer | 55 | Overall Survival | <https://www.ncbi.nlm.nih.gov/geo/query/acc.cgi?acc=GSE29013> |
| GSE17618 | Savola et al., 2011 | Normalized Expression Matrix | Sarcoma | 44 | Overall Survival | <https://www.ncbi.nlm.nih.gov/geo/query/acc.cgi?acc=GSE17618> |
| GSE21257 | Buddingh et al., 2011 | Normalized Expression Matrix | Sarcoma | 53 | Overall Survival | <https://www.ncbi.nlm.nih.gov/geo/query/acc.cgi?acc=GSE21257> |
| GSE31547 | Girard et al., 2011 | Normalized Expression Matrix | Lung Cancer | 30 | Overall Survival | <https://www.ncbi.nlm.nih.gov/geo/query/acc.cgi?acc=GSE31547> |
| GSE22541 | Wuttig et al., 2012 | Normalized Expression Matrix | Colon Adenocarcinoma | 48 | Overall Survival | <https://www.ncbi.nlm.nih.gov/geo/query/acc.cgi?acc=GSE22541> |
| GSE19234 | Bogunovic et al., 2009 | Normalized Expression Matrix | Melanoma | 44 | Overall Survival | <https://www.ncbi.nlm.nih.gov/geo/query/acc.cgi?acc=GSE19234> |
| GSE3292 | Chung et al., 2006 | Normalized Expression Matrix | Head and Neck cancer | 32 | Overall Survival | <https://www.ncbi.nlm.nih.gov/geo/query/acc.cgi?acc=GSE3292> |
| GSE16091 | Paoloni et al., 2009 | Normalized Expression Matrix | Sarcoma | 34 | Overall Survival | <https://www.ncbi.nlm.nih.gov/geo/query/acc.cgi?acc=GSE16091> |
| GSE8167 | Yamaguchi et al., 2008 | Normalized Expression Matrix | Gastrointestinal Stromal Tumor | 32 | Overall Survival | <https://www.ncbi.nlm.nih.gov/geo/query/acc.cgi?acc=GSE8167> |
| GSE5287 | Als et al., 2007 | Normalized Expression Matrix | Bladder Cancer | 30 | Overall Survival | <https://www.ncbi.nlm.nih.gov/geo/query/acc.cgi?acc=GSE5287> |
| GSE19829 | Konstantinopoulos et al., 2010 | Normalized Expression Matrix | Ovarian Cancer | 28 | Overall Survival | <https://www.ncbi.nlm.nih.gov/geo/query/acc.cgi?acc=GSE19829> |
| GSE6253 | Lu et al., 2006 | Normalized Expression Matrix | Lung Cancer | 18 | Overall Survival | <https://www.ncbi.nlm.nih.gov/geo/query/acc.cgi?acc=GSE6253> |

**Table S2**. List of Gene Signatures used. Ensembl IDs for the Signatures used in this study. Please refer to ref-13 (Thorsson et al) for the list of signatures used as pre-calculated enrichments from TCGA.

Barrier

ENSG00000143631 ENSG00000184292 ENSG00000134762 ENSG00000096696 ENSG00000118898 ENSG00000184363 ENSG00000173801 ENSG00000151914 ENSG00000136160 ENSG00000214548 ENSG00000132432 ENSG00000140950 ENSG00000075089 ENSG00000054793 ENSG00000084676 ENSG00000153944 ENSG00000198682 ENSG00000149925 ENSG00000102935 ENSG00000136960 ENSG00000168061 ENSG00000164808 ENSG00000170370 ENSG00000185697 ENSG00000165632 ENSG00000115902 ENSG00000114315 ENSG00000185559

CAF

ENSG00000078098 ENSG00000107562

Checkpoint

ENSG00000135077 ENSG00000181847 ENSG00000089692 ENSG00000107738 ENSG00000122223 ENSG00000117281 ENSG00000114455 ENSG00000204290 ENSG00000103855 ENSG00000134258 ENSG00000204577

EMT

ENSG00000166333 ENSG00000169756 ENSG00000065534 ENSG00000118260 ENSG00000162896 ENSG00000189377 ENSG00000167601 ENSG00000169071 ENSG00000114251 ENSG00000134013 ENSG00000233608 ENSG00000149591 ENSG00000078098 ENSG00000136634 ENSG00000112715 ENSG00000150630 ENSG00000108691 ENSG00000108688 ENSG00000108700 ENSG00000181374 ENSG00000006210

EMT_msigDB ENSG00000154175 ENSG00000107796 ENSG00000148848 ENSG00000166825 ENSG00000105290 ENSG00000109321 ENSG00000176788 ENSG00000176697 ENSG00000182492 ENSG00000168487 ENSG00000182985 ENSG00000122786 ENSG00000128595 ENSG00000112186 ENSG00000042493 ENSG00000026508 ENSG00000085063 ENSG00000140937 ENSG00000170558 ENSG00000113361 ENSG00000060718 ENSG00000111799 ENSG00000084636 ENSG00000108821 ENSG00000164692 ENSG00000168542 ENSG00000187498 ENSG00000134871 ENSG00000130635 ENSG00000204262 ENSG00000080573 ENSG00000142173 ENSG00000163359 ENSG00000114270 ENSG00000171812 ENSG00000105664 ENSG00000122218 ENSG00000006016 ENSG00000118523 ENSG00000164932 ENSG00000163739 ENSG00000107562 ENSG00000124875 ENSG00000142871 ENSG00000153071 ENSG00000011465 ENSG00000107984 ENSG00000113657 ENSG00000151914 ENSG00000143369 ENSG00000106823 ENSG00000164176 ENSG00000172638 ENSG00000049540 ENSG00000142227 ENSG00000111674 ENSG00000078098 ENSG00000026103 ENSG00000077942 ENSG00000163520 ENSG00000140092 ENSG00000166147 ENSG00000138829 ENSG00000073712 ENSG00000138685 ENSG00000196924 ENSG00000122176 ENSG00000115414 ENSG00000176692 ENSG00000163430 ENSG00000070404 ENSG00000179163 ENSG00000177283 ENSG00000116717 ENSG00000099860 ENSG00000180447 ENSG00000164949 ENSG00000152661 ENSG00000139278 ENSG00000130309 ENSG00000063660 ENSG00000116157 ENSG00000166923 ENSG00000166033 ENSG00000115738 ENSG00000115457 ENSG00000146674 ENSG00000141753 ENSG00000164136 ENSG00000008517 ENSG00000136244 ENSG00000169429 ENSG00000122641 ENSG00000164171 ENSG00000161638 ENSG00000138448 ENSG00000150093 ENSG00000259207 ENSG00000082781 ENSG00000177606 ENSG00000101680 ENSG00000196569 ENSG00000053747 ENSG00000135862 ENSG00000058085 ENSG00000117385 ENSG00000100097 ENSG00000113083 ENSG00000129038 ENSG00000134013 ENSG00000123384 ENSG00000172061 ENSG00000139329 ENSG00000198934 ENSG00000132561 ENSG00000132031 ENSG00000166508 ENSG00000106484 ENSG00000197614 ENSG00000111341 ENSG00000196611 ENSG00000157227 ENSG00000087245 ENSG00000149968 ENSG00000163132 ENSG00000101825 ENSG00000101335 ENSG00000065534 ENSG00000087303 ENSG00000166741 ENSG00000134250 ENSG00000135318 ENSG00000182667 ENSG00000180914 ENSG00000106333 ENSG00000163710 ENSG00000113721 ENSG00000131435 ENSG00000070087 ENSG00000011422 ENSG00000083444 ENSG00000152952 ENSG00000106397 ENSG00000124225 ENSG00000109099 ENSG00000133110 ENSG00000166794 ENSG00000116132 ENSG00000275896 ENSG00000087494 ENSG00000163661 ENSG00000073008 ENSG00000116260 ENSG00000117152 ENSG00000143878 ENSG00000130066 ENSG00000171951 ENSG00000115884 ENSG00000124145 ENSG00000106366 ENSG00000135919 ENSG00000149257 ENSG00000104332 ENSG00000106483 ENSG00000163069 ENSG00000170624 ENSG00000102683 ENSG00000130821 ENSG00000145147 ENSG00000184347 ENSG00000019549 ENSG00000172164 ENSG00000113140 ENSG00000152377 ENSG00000118785 ENSG00000149591 ENSG00000105825 ENSG00000105329 ENSG00000120708 ENSG00000069702 ENSG00000198959 ENSG00000137801 ENSG00000186340 ENSG00000154096 ENSG00000102265 ENSG00000100234 ENSG00000041982 ENSG00000118503 ENSG00000164761 ENSG00000006327 ENSG00000140416 ENSG00000198467 ENSG00000167460 ENSG00000162692 ENSG00000038427 ENSG00000112715 ENSG00000150630 ENSG00000026025 ENSG00000115935 ENSG00000114251

Hypoxia

ENSG00000128271 ENSG00000138185 ENSG00000135318

ICD

ENSG00000138185 ENSG00000179218 ENSG00000123384 ENSG00000125538 ENSG00000112115 ENSG00000137462 ENSG00000164342 ENSG00000136869 ENSG00000172936 ENSG00000185507

ICR

ENSG00000271503 ENSG00000138755 ENSG00000169245 ENSG00000111537 ENSG00000073861 ENSG00000153563 ENSG00000172116 ENSG00000125347 ENSG00000113302 ENSG00000115415 ENSG00000180644 ENSG00000145649 ENSG00000100453 ENSG00000100450 ENSG00000115523 ENSG00000163599 ENSG00000188389 ENSG00000120217 ENSG00000049768 ENSG00000131203

IL17 ENSG00000125571 ENSG00000197272 ENSG00000112115 ENSG00000136695 ENSG00000136697 ENSG00000112116 ENSG00000185507 ENSG00000113302 ENSG00000232810 ENSG00000110944 ENSG00000168811 ENSG00000136694 ENSG00000136244 ENSG00000111537 ENSG00000115009 ENSG00000112486 ENSG00000163739 ENSG00000163734 ENSG00000163735 ENSG00000124875 ENSG00000169429 ENSG00000149968 ENSG00000143365 ENSG00000118515 ENSG00000056972 ENSG00000138684 ENSG00000127318 ENSG00000239732 ENSG00000168610 ENSG00000110092 ENSG00000105329 ENSG00000120659 ENSG00000090339 ENSG00000125538 ENSG00000115594 ENSG00000136689 ENSG00000164400 ENSG00000127318 ENSG00000100985 ENSG00000007171 ENSG00000115590 ENSG00000172936 ENSG00000112115 ENSG00000143365 ENSG00000106178 ENSG00000162594 ENSG00000127743

LAP-Helios

ENSG00000030419 ENSG00000137507 ENSG00000105329

MAPK

ENSG00000149930 ENSG00000141510 ENSG00000102882 ENSG00000095015 ENSG00000186868 ENSG00000204389 ENSG00000136068 ENSG00000135090 ENSG00000167193 ENSG00000071242 ENSG00000065559 ENSG00000120129 ENSG00000157388 ENSG00000107643 ENSG00000172575 ENSG00000006283

Bcell

ENSG00000105369 ENSG00000110777 ENSG00000152256 ENSG00000057657 ENSG00000159958 ENSG00000048462 ENSG00000004468 ENSG00000137101 ENSG00000211899 ENSG00000128322

Desmoplastic_Stroma

ENSG00000108821 ENSG00000164692 ENSG00000168542 ENSG00000130635 ENSG00000204262 ENSG00000144810 ENSG00000171812 ENSG00000123500 ENSG00000111799 ENSG00000084636 ENSG00000113721 ENSG00000078098 ENSG00000072682 ENSG00000087245 ENSG00000129038

Invasive_epithelium

ENSG00000185479 ENSG00000108244 ENSG00000169715 ENSG00000198417 ENSG00000205364 ENSG00000260549 ENSG00000205358 ENSG00000169688 ENSG00000205362

Tcell

ENSG00000116824 ENSG00000167286 ENSG00000134460 ENSG00000100385 ENSG00000147168 ENSG00000010610 ENSG00000153563 ENSG00000172116 ENSG00000145649 ENSG00000100453 ENSG00000113088 ENSG00000100450 ENSG00000115415 ENSG00000138378 ENSG00000185215 ENSG00000145779

NFKB

ENSG00000117228 ENSG00000204264 ENSG00000240065 ENSG00000125347 ENSG00000271503 ENSG00000168394 ENSG00000118503 ENSG00000008517 ENSG00000111252 ENSG00000146232

NOS

ENSG00000089250 ENSG00000007171 ENSG00000164867

PI3Kgamma

ENSG00000197461 ENSG00000100311 ENSG00000113070 ENSG00000122861 ENSG00000117525 ENSG00000185745 ENSG00000103855 ENSG00000136244 ENSG00000108688 ENSG00000137331

PPARG

ENSG00000132170 ENSG00000186350

PPP2CA

ENSG00000113575

SGK1

ENSG00000118515 ENSG00000105723 ENSG00000082701 ENSG00000105329 ENSG00000113520

SHC1_pSTAT3 ENSG00000168610 ENSG00000134352 ENSG00000109320 ENSG00000075426 ENSG00000033867 ENSG00000148154 ENSG00000143384 ENSG00000170525 ENSG00000096968 ENSG00000145780 ENSG00000020577 ENSG00000241978 ENSG00000131016 ENSG00000148175 ENSG00000090339 ENSG00000136068 ENSG00000139793 ENSG00000196136 ENSG00000092969 ENSG00000112096 ENSG00000137801 ENSG00000113916 ENSG00000125730 ENSG00000127951 ENSG00000120156 ENSG00000178726 ENSG00000185499 ENSG00000141458 ENSG00000053747 ENSG00000131459 ENSG00000130164 ENSG00000162407 ENSG00000139946 ENSG00000118971 ENSG00000171564 ENSG00000129988 ENSG00000135744 ENSG00000175899 ENSG00000173334 ENSG00000173262 ENSG00000087245 ENSG00000184557 ENSG00000162772 ENSG00000059804 ENSG00000170345 ENSG00000171557 ENSG00000128016 ENSG00000172059 ENSG00000152952 ENSG00000136244

STING_IRF3

ENSG00000126456 ENSG00000164430 ENSG00000123685 ENSG00000178726 ENSG00000140968 ENSG00000197992 ENSG00000083457

TAM

ENSG00000092445 ENSG00000167601 ENSG00000153208

Th2

ENSG00000125538 ENSG00000027697 ENSG00000159128 ENSG00000168610 ENSG00000243646 ENSG00000077238 ENSG00000166888 ENSG00000183625 ENSG00000137077 ENSG00000136634 ENSG00000105329 ENSG00000113520 ENSG00000169194 ENSG00000113525 ENSG00000102970 ENSG00000102962 ENSG00000049768 ENSG00000126353 ENSG00000198851 ENSG00000102245 ENSG00000172724 ENSG00000183813 ENSG00000107485

Tolerogenic DCs

ENSG00000135218 ENSG00000145850 ENSG00000138448

WNT

ENSG00000108947 ENSG00000115266 ENSG00000081059 ENSG00000136997 ENSG00000140262 ENSG00000112715

**Table S3**. Ensembl Gene IDs that correlate significantly with ICR in ICR-Enabled (605 IDs) or ICR disabled (307 IDs) cohorts.

ICR_Enabled

ENSG00000135426 ENSG00000185862 ENSG00000134539 ENSG00000150637 ENSG00000110876 ENSG00000072818 ENSG00000140368 ENSG00000164691 ENSG00000188404 ENSG00000049768 ENSG00000102524 ENSG00000259834 ENSG00000211727 ENSG00000242258 ENSG00000127951 ENSG00000180549 ENSG00000205220 ENSG00000187862 ENSG00000166501 ENSG00000168394 ENSG00000137101 ENSG00000163219 ENSG00000004468 ENSG00000211767 ENSG00000167077 ENSG00000211809 ENSG00000276819 ENSG00000140968 ENSG00000211771 ENSG00000227039 ENSG00000138378 ENSG00000166523 ENSG00000276405 ENSG00000104814 ENSG00000167664 ENSG00000150681 ENSG00000261644 ENSG00000211816 ENSG00000213809 ENSG00000110031 ENSG00000167984 ENSG00000188011 ENSG00000159753 ENSG00000230099 ENSG00000166710 ENSG00000100079 ENSG00000196329 ENSG00000180096 ENSG00000111729 ENSG00000204261 ENSG00000181631 ENSG00000211725 ENSG00000177272 ENSG00000213203 ENSG00000002549 ENSG00000160219 ENSG00000133561 ENSG00000077420 ENSG00000261416 ENSG00000106948 ENSG00000236481 ENSG00000101347 ENSG00000251578 ENSG00000074706 ENSG00000106560 ENSG00000121895 ENSG00000168062 ENSG00000111801 ENSG00000257924 ENSG00000204264 ENSG00000211714 ENSG00000211818 ENSG00000156587 ENSG00000175779 ENSG00000198771 ENSG00000107742 ENSG00000139626 ENSG00000229769 ENSG00000229474 ENSG00000179840 ENSG00000255569 ENSG00000276070 ENSG00000138684 ENSG00000134470 ENSG00000102096 ENSG00000240403 ENSG00000182578 ENSG00000128438 ENSG00000198286 ENSG00000197992 ENSG00000206503 ENSG00000255819 ENSG00000267121 ENSG00000118308 ENSG00000186470 ENSG00000253409 ENSG00000173193 ENSG00000204267 ENSG00000243772 ENSG00000174600 ENSG00000124334 ENSG00000170128 ENSG00000180448 ENSG00000185880 ENSG00000211749 ENSG00000023445 ENSG00000188282 ENSG00000268804 ENSG00000167633 ENSG00000105383 ENSG00000204161 ENSG00000028277 ENSG00000280153 ENSG00000240954 ENSG00000204525 ENSG00000140105 ENSG00000237914 ENSG00000136573 ENSG00000279805 ENSG00000085514 ENSG00000107099 ENSG00000119917 ENSG00000198574 ENSG00000277632 ENSG00000211813 ENSG00000222037 ENSG00000167261 ENSG00000166527 ENSG00000276231 ENSG00000185669 ENSG00000240350 ENSG00000198223 ENSG00000124196 ENSG00000211593 ENSG00000107551 ENSG00000115267 ENSG00000239636 ENSG00000087589 ENSG00000104970 ENSG00000261218 ENSG00000278195 ENSG00000233999 ENSG00000239819 ENSG00000225864 ENSG00000160683 ENSG00000254087 ENSG00000240143 ENSG00000168071 ENSG00000259337 ENSG00000125498 ENSG00000101445 ENSG00000235532 ENSG00000102794 ENSG00000198178 ENSG00000211665 ENSG00000185433 ENSG00000230266 ENSG00000237111 ENSG00000214212 ENSG00000183542 ENSG00000211935 ENSG00000278982 ENSG00000179921 ENSG00000115935 ENSG00000205809 ENSG00000234184 ENSG00000146070 ENSG00000280194 ENSG00000064201 ENSG00000249096 ENSG00000171115 ENSG00000253838 ENSG00000011590 ENSG00000211667 ENSG00000244273 ENSG00000108405 ENSG00000258875 ENSG00000261448 ENSG00000177807 ENSG00000211676 ENSG00000155629 ENSG00000254056 ENSG00000275158 ENSG00000101017 ENSG00000228863 ENSG00000278857 ENSG00000253291 ENSG00000273445 ENSG00000254287 ENSG00000126246 ENSG00000121380 ENSG00000078596 ENSG00000271178 ENSG00000278263 ENSG00000230138 ENSG00000248099 ENSG00000251546 ENSG00000083454 ENSG00000253132 ENSG00000145779 ENSG00000056558 ENSG00000271130 ENSG00000259747 ENSG00000262370 ENSG00000270472 ENSG00000211720 ENSG00000211647 ENSG00000225783 ENSG00000253545 ENSG00000229228 ENSG00000116701 ENSG00000276597 ENSG00000269220 ENSG00000107738 ENSG00000224666 ENSG00000135439 ENSG00000188403 ENSG00000197476 ENSG00000259092 ENSG00000180539 ENSG00000267046 ENSG00000271375 ENSG00000211904 ENSG00000137959 ENSG00000134326 ENSG00000007264 ENSG00000236320 ENSG00000279396 ENSG00000162711 ENSG00000232578 ENSG00000198624 ENSG00000270379 ENSG00000260828 ENSG00000068079 ENSG00000068831 ENSG00000253120 ENSG00000261435 ENSG00000242580 ENSG00000234389 ENSG00000167483 ENSG00000233387 ENSG00000279082 ENSG00000236946 ENSG00000119922 ENSG00000211695 ENSG00000182557 ENSG00000187796 ENSG00000253239 ENSG00000253742 ENSG00000049247 ENSG00000274536 ENSG00000253234 ENSG00000253497 ENSG00000242048 ENSG00000253709 ENSG00000241560 ENSG00000237398 ENSG00000224220 ENSG00000127152 ENSG00000259436 ENSG00000081320 ENSG00000242766 ENSG00000254174 ENSG00000136869 ENSG00000230481 ENSG00000253883 ENSG00000182179 ENSG00000211911 ENSG00000137965 ENSG00000065675 ENSG00000163518 ENSG00000231858 ENSG00000277282 ENSG00000211672 ENSG00000229613 ENSG00000253818 ENSG00000175841 ENSG00000211924 ENSG00000254098 ENSG00000259680 ENSG00000184371 ENSG00000181215 ENSG00000134594 ENSG00000197549 ENSG00000239571 ENSG00000152207 ENSG00000253988 ENSG00000278473 ENSG00000137491 ENSG00000253490 ENSG00000171608 ENSG00000116663 ENSG00000111913 ENSG00000231331 ENSG00000231621 ENSG00000217482 ENSG00000008516 ENSG00000254046 ENSG00000141497 ENSG00000122043 ENSG00000205056 ENSG00000136514 ENSG00000270864 ENSG00000223511 ENSG00000235366 ENSG00000249454 ENSG00000197142 ENSG00000273824 ENSG00000106565 ENSG00000248898 ENSG00000183484 ENSG00000253451 ENSG00000274508 ENSG00000123685 ENSG00000224607 ENSG00000211905 ENSG00000089127 ENSG00000242534 ENSG00000204136 ENSG00000267654 ENSG00000239975 ENSG00000198846 ENSG00000248993 ENSG00000176160 ENSG00000211633 ENSG00000188848 ENSG00000234515 ENSG00000237470 ENSG00000205810 ENSG00000115604 ENSG00000255163 ENSG00000092929 ENSG00000253957 ENSG00000256128 ENSG00000158473 ENSG00000137474 ENSG00000069424 ENSG00000233732 ENSG00000232613 ENSG00000248571 ENSG00000197794 ENSG00000142583 ENSG00000230747 ENSG00000225885 ENSG00000189238 ENSG00000248991 ENSG00000253481 ENSG00000132530 ENSG00000211978 ENSG00000117594 ENSG00000122025 ENSG00000204670 ENSG00000237372 ENSG00000260314 ENSG00000232884 ENSG00000236790 ENSG00000211931 ENSG00000181074 ENSG00000261704 ENSG00000224585 ENSG00000169313 ENSG00000259772 ENSG00000253209 ENSG00000253441 ENSG00000163492 ENSG00000211645 ENSG00000109861 ENSG00000275418 ENSG00000234332 ENSG00000257221 ENSG00000151651 ENSG00000211654 ENSG00000253522 ENSG00000253247 ENSG00000253460 ENSG00000152784 ENSG00000225756 ENSG00000270187 ENSG00000165949 ENSG00000256590 ENSG00000254228 ENSG00000177989 ENSG00000234956 ENSG00000150045 ENSG00000165457 ENSG00000258572 ENSG00000242324 ENSG00000102970 ENSG00000054219 ENSG00000237592 ENSG00000170819 ENSG00000279149 ENSG00000128218 ENSG00000218052 ENSG00000161944 ENSG00000254326 ENSG00000132832 ENSG00000270318 ENSG00000250274 ENSG00000002933 ENSG00000227421 ENSG00000117226 ENSG00000112486 ENSG00000223750 ENSG00000255197 ENSG00000138642 ENSG00000240040 ENSG00000253359 ENSG00000211887 ENSG00000170396 ENSG00000138646 ENSG00000105609 ENSG00000135905 ENSG00000230006 ENSG00000167476 ENSG00000241666 ENSG00000186188 ENSG00000253763 ENSG00000118849 ENSG00000233308 ENSG00000196169 ENSG00000241678 ENSG00000042980 ENSG00000250155 ENSG00000165409 ENSG00000116852 ENSG00000261834 ENSG00000254157 ENSG00000268758 ENSG00000214872 ENSG00000263417 ENSG00000249806 ENSG00000229664 ENSG00000276085 ENSG00000164512 ENSG00000123146 ENSG00000172575 ENSG00000269800 ENSG00000115155 ENSG00000211923 ENSG00000189350 ENSG00000129226 ENSG00000128604 ENSG00000271581 ENSG00000241163 ENSG00000259954 ENSG00000226806 ENSG00000211979 ENSG00000124721 ENSG00000232591 ENSG00000254176 ENSG00000234142 ENSG00000217643 ENSG00000223662 ENSG00000231964 ENSG00000253435 ENSG00000157873 ENSG00000180828 ENSG00000225948 ENSG00000227017 ENSG00000246223 ENSG00000237943 ENSG00000272463 ENSG00000270467 ENSG00000228037 ENSG00000255354 ENSG00000227678 ENSG00000163121 ENSG00000179331 ENSG00000230521 ENSG00000164112 ENSG00000081985 ENSG00000048740 ENSG00000112818 ENSG00000164400 ENSG00000073737 ENSG00000247193 ENSG00000254521 ENSG00000218565 ENSG00000265118 ENSG00000140090 ENSG00000105409 ENSG00000257242 ENSG00000131142 ENSG00000259225 ENSG00000092345 ENSG00000120337 ENSG00000197880 ENSG00000160326 ENSG00000221887 ENSG00000258512 ENSG00000226738 ENSG00000153789 ENSG00000130475 ENSG00000205436 ENSG00000270164 ENSG00000172156 ENSG00000137731 ENSG00000158525 ENSG00000164308 ENSG00000036565 ENSG00000232810 ENSG00000204632 ENSG00000241134 ENSG00000111732 ENSG00000177699 ENSG00000253796 ENSG00000172794 ENSG00000256862 ENSG00000249896 ENSG00000241220 ENSG00000130203 ENSG00000185745 ENSG00000132744 ENSG00000135925 ENSG00000233665 ENSG00000266378 ENSG00000211880 ENSG00000227766 ENSG00000161640 ENSG00000204622 ENSG00000113303 ENSG00000256714 ENSG00000225541 ENSG00000253535 ENSG00000157368 ENSG00000225851 ENSG00000146374 ENSG00000176177 ENSG00000259641 ENSG00000148734 ENSG00000172752 ENSG00000168811 ENSG00000163687 ENSG00000205837 ENSG00000276778 ENSG00000124557 ENSG00000259922 ENSG00000202533 ENSG00000258521 ENSG00000162594 ENSG00000121858 ENSG00000137571 ENSG00000159958 ENSG00000113249 ENSG00000156219 ENSG00000111863 ENSG00000244649 ENSG00000160307 ENSG00000238057 ENSG00000186891 ENSG00000205755 ENSG00000260302 ENSG00000105492 ENSG00000167912 ENSG00000258268 ENSG00000187510 ENSG00000166035 ENSG00000257275 ENSG00000179088 ENSG00000227591 ENSG00000172005 ENSG00000162706 ENSG00000106178 ENSG00000146666 ENSG00000205038 ENSG00000178860 ENSG00000268916 ENSG00000136541 ENSG00000144837 ENSG00000203756 ENSG00000254211 ENSG00000254036 ENSG00000147647 ENSG00000226197 ENSG00000225107 ENSG00000229056 ENSG00000263961 ENSG00000253891 ENSG00000143196 ENSG00000213512 ENSG00000176320 ENSG00000196188 ENSG00000233473 ENSG00000182566 ENSG00000183549 ENSG00000111339 ENSG00000143452 ENSG00000236977 ENSG00000163739 ENSG00000172927 ENSG00000223930 ENSG00000174325 ENSG00000204866 ENSG00000172236 ENSG00000109511 ENSG00000022556 ENSG00000094755 ENSG00000160183 ENSG00000196616 ENSG00000232498 ENSG00000129988 ENSG00000162896 ENSG00000174776 ENSG00000227403

ICR_Disabled

ENSG00000140030 ENSG00000171700 ENSG00000152213 ENSG00000038945 ENSG00000183486 ENSG00000131378 ENSG00000196843 ENSG00000140379 ENSG00000184497 ENSG00000278231 ENSG00000102445 ENSG00000127507 ENSG00000254288 ENSG00000100097 ENSG00000171522 ENSG00000060982 ENSG00000005059 ENSG00000254503 ENSG00000161638 ENSG00000188015 ENSG00000125810 ENSG00000180871 ENSG00000134242 ENSG00000245848 ENSG00000260228 ENSG00000116774 ENSG00000164687 ENSG00000121552 ENSG00000184557 ENSG00000162493 ENSG00000134955 ENSG00000246100 ENSG00000182511 ENSG00000214787 ENSG00000124731 ENSG00000106066 ENSG00000095970 ENSG00000106823 ENSG00000011422 ENSG00000152760 ENSG00000197405 ENSG00000231560 ENSG00000099860 ENSG00000115107 ENSG00000108950 ENSG00000115414 ENSG00000106366 ENSG00000234964 ENSG00000184106 ENSG00000115232 ENSG00000136689 ENSG00000143333 ENSG00000134802 ENSG00000266913 ENSG00000250771 ENSG00000026508 ENSG00000120457 ENSG00000117643 ENSG00000148680 ENSG00000204971 ENSG00000188001 ENSG00000188060 ENSG00000133055 ENSG00000125430 ENSG00000117009 ENSG00000111341 ENSG00000158270 ENSG00000145703 ENSG00000234147 ENSG00000174807 ENSG00000011028 ENSG00000173110 ENSG00000132205 ENSG00000142089 ENSG00000135842 ENSG00000231752 ENSG00000167600 ENSG00000139211 ENSG00000159216 ENSG00000164047 ENSG00000107485 ENSG00000135074 ENSG00000163064 ENSG00000123342 ENSG00000197769 ENSG00000258227 ENSG00000114115 ENSG00000018280 ENSG00000226329 ENSG00000169071 ENSG00000136231 ENSG00000131435 ENSG00000124491 ENSG00000136235 ENSG00000134668 ENSG00000081059 ENSG00000115380 ENSG00000102359 ENSG00000263155 ENSG00000179428 ENSG00000121933 ENSG00000257336 ENSG00000138435 ENSG00000138080 ENSG00000132386 ENSG00000163661 ENSG00000029559 ENSG00000121570 ENSG00000268223 ENSG00000177294 ENSG00000255189 ENSG00000139610 ENSG00000236700 ENSG00000253557 ENSG00000171812 ENSG00000189068 ENSG00000267123 ENSG00000257345 ENSG00000089327 ENSG00000147065 ENSG00000107562 ENSG00000171223 ENSG00000279447 ENSG00000148848 ENSG00000126337 ENSG00000257838 ENSG00000064300 ENSG00000170801 ENSG00000014257 ENSG00000222041 ENSG00000266709 ENSG00000137441 ENSG00000168398 ENSG00000241644 ENSG00000196136 ENSG00000104856 ENSG00000172216 ENSG00000106483 ENSG00000144681 ENSG00000160013 ENSG00000158104 ENSG00000164220 ENSG00000137976 ENSG00000163221 ENSG00000177363 ENSG00000149380 ENSG00000226822 ENSG00000197093 ENSG00000175084 ENSG00000131355 ENSG00000237927 ENSG00000135046 ENSG00000079931 ENSG00000087245 ENSG00000110719 ENSG00000124216 ENSG00000188488 ENSG00000126467 ENSG00000187498 ENSG00000187955 ENSG00000115457 ENSG00000102265 ENSG00000006606 ENSG00000183644 ENSG00000257219 ENSG00000182782 ENSG00000128510 ENSG00000125538 ENSG00000004776 ENSG00000164932 ENSG00000255750 ENSG00000164692 ENSG00000169436 ENSG00000130635 ENSG00000175592 ENSG00000112319 ENSG00000273259 ENSG00000122861 ENSG00000099994 ENSG00000166741 ENSG00000128591 ENSG00000168542 ENSG00000172935 ENSG00000259616 ENSG00000226808 ENSG00000104415 ENSG00000163464 ENSG00000170439 ENSG00000103490 ENSG00000144810 ENSG00000248323 ENSG00000092969 ENSG00000129009 ENSG00000233521 ENSG00000136315 ENSG00000163017 ENSG00000229588 ENSG00000183671 ENSG00000198848 ENSG00000204403 ENSG00000183844 ENSG00000123689 ENSG00000151790 ENSG00000224164 ENSG00000234754 ENSG00000163359 ENSG00000156966 ENSG00000108821 ENSG00000144481 ENSG00000122176 ENSG00000196503 ENSG00000205362 ENSG00000275395 ENSG00000004948 ENSG00000042493 ENSG00000224184 ENSG00000139329 ENSG00000222047 ENSG00000231971 ENSG00000147614 ENSG00000147206 ENSG00000188056 ENSG00000138061 ENSG00000183019 ENSG00000142173 ENSG00000147434 ENSG00000179639 ENSG00000155011 ENSG00000198768 ENSG00000106333 ENSG00000123496 ENSG00000137868 ENSG00000170290 ENSG00000145358 ENSG00000234380 ENSG00000106624 ENSG00000168779 ENSG00000180785 ENSG00000134871 ENSG00000107796 ENSG00000148735 ENSG00000175262 ENSG00000231817 ENSG00000115008 ENSG00000158710 ENSG00000176014 ENSG00000204291 ENSG00000148926 ENSG00000112299 ENSG00000166482 ENSG00000248227 ENSG00000171864 ENSG00000069399 ENSG00000134198 ENSG00000171101 ENSG00000272632 ENSG00000171819 ENSG00000133110 ENSG00000172061 ENSG00000184344 ENSG00000162777 ENSG00000225649 ENSG00000279965 ENSG00000164294 ENSG00000233968 ENSG00000102802 ENSG00000198542 ENSG00000266835 ENSG00000138166 ENSG00000163792 ENSG00000130176 ENSG00000224389 ENSG00000253123 ENSG00000169397 ENSG00000134817 ENSG00000074966 ENSG00000164920 ENSG00000125355 ENSG00000137801 ENSG00000143768 ENSG00000214688 ENSG00000141526 ENSG00000013588 ENSG00000109610 ENSG00000174500 ENSG00000164761 ENSG00000179593 ENSG00000166670 ENSG00000105991 ENSG00000128342 ENSG00000112936 ENSG00000200378 ENSG00000135914 ENSG00000179097 ENSG00000104055 ENSG00000212206 ENSG00000260802 ENSG00000111424 ENSG00000104518 ENSG00000240602 ENSG00000227992 ENSG00000255443 ENSG00000006327 ENSG00000127472 ENSG00000254337 ENSG00000168140 ENSG00000106809 ENSG00000134215 ENSG00000205364 ENSG00000157613 ENSG00000233730 ENSG00000224995 ENSG00000159167 ENSG00000073734 ENSG00000167332 ENSG00000178602 ENSG00000108846 ENSG00000160505 ENSG00000164694 ENSG00000133067 ENSG00000109113 ENSG00000249001 ENSG00000163520 ENSG00000174343 ENSG00000180638 ENSG00000167244 ENSG00000149451 ENSG00000225511 ENSG00000197632 ENSG00000116785 ENSG00000196611 ENSG00000154646 ENSG00000129038 ENSG00000164283 ENSG00000109321 ENSG00000130600 ENSG00000158022 ENSG00000149968 ENSG00000148204 ENSG00000198099 ENSG00000175664 ENSG00000182742 ENSG00000231991 ENSG00000231298 ENSG00000139330 ENSG00000233539 ENSG00000004846 ENSG00000258754 ENSG00000171540 ENSG00000136542 ENSG00000275830 ENSG00000130513 ENSG00000198796 ENSG00000106538 ENSG00000124102 ENSG00000171658 ENSG00000198074 ENSG00000128714 ENSG00000244681 ENSG00000184937 ENSG00000183742 ENSG00000205835 ENSG00000135903 ENSG00000172020 ENSG00000149418 ENSG00000173917 ENSG00000133636 ENSG00000145824 ENSG00000106511 ENSG00000137745 ENSG00000253293 ENSG00000172023 ENSG00000183798 ENSG00000106004 ENSG00000115386 ENSG00000140067 ENSG0000012

**Table S4.** Hazard Ratios and p-values (same as Figure 1C but using the samples not used in MIRACLE feature selection (6294 TCGA samples)). Comparative analysis of immune-related signatures from various sources. The tables show coxph statistics using the samples that are not used in the Miracle Feature selection (ie. training set excluded). Signatures associated with better-OS and worse-OS (B) are shown.

Table S4A

| **Predictor** | **coxph_coef** | **pval** | **Hazard_Ratio** | **Conf_lower** | **Conf_Upper** |
| --- | --- | --- | --- | --- | --- |
| Miracle | -2.50713 | 2.60E-13 | 0.081502 | 0.041627 | 0.159575 |
| Th17.Cells+ | -0.51295 | 8.69E-07 | 0.598728 | 0.488056 | 0.734494 |
| Lymphocytes+ | -0.68373 | 2.64E-05 | 0.504733 | 0.366923 | 0.694302 |
| Mast.C.Rest+ | -1.2534 | 0.000505 | 0.285533 | 0.140896 | 0.578649 |
| Lymph.Infilt.Sig.Sc+ | -0.30288 | 0.003976 | 0.738689 | 0.6011 | 0.907771 |
| ICR* | -0.29067 | 0.007662 | 0.747761 | 0.60392 | 0.925863 |
| CD4.T.Mem.Act+ | -0.63383 | 0.007763 | 0.530557 | 0.332716 | 0.846038 |
| Tcell* | -0.30086 | 0.009576 | 0.740181 | 0.589508 | 0.929366 |
| TIL.Reg.Frac+ | -0.73435 | 0.012092 | 0.479816 | 0.270385 | 0.851465 |
| Nonsil.Mut.Rate+ | -4.61643 | 0.014319 | 0.009888 | 0.000246 | 0.397688 |
| Bcell* | -0.28074 | 0.019959 | 0.755223 | 0.596193 | 0.956673 |
| Sil.Mut.Rate+ | -4.1015 | 0.025218 | 0.016548 | 0.000456 | 0.600729 |
| TCR.Shannon+ | -0.38663 | 0.03615 | 0.679342 | 0.473171 | 0.975348 |
| TCR.Evenness+ | -0.77964 | 0.039652 | 0.458572 | 0.218194 | 0.963766 |
| DC.Resting+ | -0.70315 | 0.056704 | 0.495023 | 0.24018 | 1.020268 |
| CD4.T.Cells+ | -0.37795 | 0.05691 | 0.685265 | 0.464401 | 1.011171 |
| CD4.T.Mem.Rest+ | -0.31302 | 0.085755 | 0.731235 | 0.511668 | 1.045023 |
| NK.C.Act+ | -0.48118 | 0.087251 | 0.618054 | 0.356055 | 1.072842 |
| SNV.Neoantigens+ | -2.67123 | 0.111057 | 0.069167 | 0.002588 | 1.848543 |
| T.Cells.FH+ | -0.31456 | 0.123645 | 0.730111 | 0.489197 | 1.089666 |
| Tregs+ | -0.32256 | 0.183977 | 0.724295 | 0.450052 | 1.16565 |
| MAPK* | -0.39867 | 0.191295 | 0.671209 | 0.36912 | 1.22053 |
| Monocytes+ | -0.33687 | 0.231201 | 0.714005 | 0.411345 | 1.239356 |
| TCR.Richness+ | -1.07455 | 0.245151 | 0.341451 | 0.05576 | 2.090887 |
| BCR.Richness+ | -0.60089 | 0.262864 | 0.548326 | 0.191523 | 1.569843 |
| B.Cells.Naive+ | -0.23772 | 0.313238 | 0.788426 | 0.49672 | 1.25144 |
| Th2* | -0.17945 | 0.316769 | 0.835726 | 0.588142 | 1.187531 |
| CHCKP* | -0.1673 | 0.322435 | 0.845944 | 0.607321 | 1.178326 |
| DCs+ | -0.23111 | 0.349878 | 0.793651 | 0.488868 | 1.288449 |
| Plasma.Cells+ | -0.20256 | 0.382372 | 0.816639 | 0.518382 | 1.286501 |
| PPARG* | -0.08773 | 0.514078 | 0.916005 | 0.703797 | 1.192197 |
| CD4.T.Naive+ | -0.24288 | 0.559474 | 0.784364 | 0.346959 | 1.773194 |
| Sting_IRF3* | -0.09951 | 0.570147 | 0.905283 | 0.642126 | 1.276288 |
| T.Cells.GD+ | -0.28522 | 0.589537 | 0.75185 | 0.266767 | 2.118999 |
| IFNG.Resp+ | -0.0474 | 0.620353 | 0.953705 | 0.790609 | 1.150447 |
| ICD* | -0.07077 | 0.700638 | 0.931675 | 0.649494 | 1.336453 |
| B.Cells.Memory+ | -0.07038 | 0.863794 | 0.932038 | 0.417066 | 2.082872 |
| Macroph.Reg+ | -0.00629 | 0.940871 | 0.993731 | 0.841586 | 1.17338 |
| Macrophages.M1+ | -0.01227 | 0.949612 | 0.987807 | 0.675185 | 1.445178 |
| BCR.Evenness+ | -0.00837 | 0.973269 | 0.991666 | 0.607815 | 1.617929 |

Table S4B:

| **Predictor** | **coxph_coef** | **pval** | **Hazard_Ratio** | **Conf_lower** | **Conf_Upper** |
| --- | --- | --- | --- | --- | --- |
| Proliferation+ | 0.663635 | 1.20E-17 | 1.941838 | 1.667901 | 2.260766 |
| EMT_msigdb* | 1.238755 | 7.42E-13 | 3.451314 | 2.460111 | 4.841884 |
| Wound.Healing+ | 1.000974 | 4.24E-12 | 2.72093 | 2.049997 | 3.611447 |
| TGF.b.Resp+ | 0.646765 | 2.27E-11 | 1.909355 | 1.579653 | 2.307872 |
| Th2.Cells+ | 0.778959 | 3.61E-11 | 2.179204 | 1.730323 | 2.744533 |
| Des_stroma* | 0.666608 | 1.77E-10 | 1.94762 | 1.586952 | 2.390256 |
| PI3Kgamma* | 0.974779 | 2.04E-10 | 2.650582 | 1.962666 | 3.579612 |
| Macrophages.M0+ | 0.921169 | 8.40E-08 | 2.512226 | 1.793599 | 3.518779 |
| EMT* | 0.850251 | 9.75E-07 | 2.340234 | 1.665161 | 3.288986 |
| Mast.C.Act+ | 1.429959 | 1.03E-06 | 4.178528 | 2.354433 | 7.415839 |
| Invasive_epi* | 0.555906 | 3.77E-06 | 1.74352 | 1.377467 | 2.20685 |
| CAP* | 0.38812 | 8.81E-06 | 1.474206 | 1.242305 | 1.749397 |
| CTA.Score+ | 0.709786 | 1.51E-05 | 2.033556 | 1.474516 | 2.804547 |
| SHC1* | 0.858662 | 3.95E-05 | 2.36 | 1.567141 | 3.553987 |
| Macrophages+ | 0.642027 | 0.000139 | 1.900328 | 1.365829 | 2.643996 |
| Neutrophils+ | 1.604676 | 0.000499 | 4.976246 | 2.016314 | 12.28133 |
| N.of.Segments+ | 1.079496 | 0.000688 | 2.943196 | 1.578045 | 5.489324 |
| Barrier* | 1.366045 | 0.001395 | 3.919818 | 1.695926 | 9.059931 |
| WNT* | 0.633938 | 0.00188 | 1.88502 | 1.263945 | 2.811276 |
| Aneuploidy.Score+ | 0.380932 | 0.001889 | 1.463648 | 1.15102 | 1.861189 |
| LAP_Helios* | 0.434774 | 0.003379 | 1.544614 | 1.154926 | 2.065788 |
| Fraction.Altered+ | 0.248372 | 0.004697 | 1.281937 | 1.079158 | 1.522819 |
| TAM* | 0.415126 | 0.005171 | 1.514562 | 1.13218 | 2.02609 |
| HR.Defects+ | 0.460139 | 0.006876 | 1.584295 | 1.134809 | 2.211816 |
| Tumor.Heterogen+ | 0.375266 | 0.009962 | 1.455378 | 1.094034 | 1.936069 |
| PPP2CA* | 0.290786 | 0.011058 | 1.337478 | 1.068738 | 1.673794 |
| Th1.Cells+ | 0.186461 | 0.016087 | 1.204977 | 1.035232 | 1.402555 |
| SGK1* | 0.471606 | 0.018555 | 1.602566 | 1.082205 | 2.373136 |
| Tol_DCs* | 0.288791 | 0.023204 | 1.334813 | 1.040239 | 1.712804 |
| Leuk.Frac+ | 0.299944 | 0.063881 | 1.349783 | 0.982837 | 1.85373 |
| IL17* | 0.399214 | 0.076985 | 1.490652 | 0.957689 | 2.320214 |
| Eosinophils+ | 0.600684 | 0.111953 | 1.823366 | 0.869347 | 3.824322 |
| NK.C.Rest+ | 0.275677 | 0.170585 | 1.317423 | 0.888144 | 1.95419 |
| Stroma.Frac+ | 0.144028 | 0.252438 | 1.154916 | 0.902459 | 1.477997 |
| Mast.Cells+ | 0.255382 | 0.376095 | 1.290954 | 0.733354 | 2.272522 |
| Hypoxia* | 0.073548 | 0.561766 | 1.076321 | 0.839544 | 1.379875 |
| NOS* | 0.061792 | 0.59367 | 1.063741 | 0.847718 | 1.334812 |
| Macrophages.M2+ | 0.083389 | 0.641349 | 1.086964 | 0.765306 | 1.543815 |
| DC.Act+ | 0.064662 | 0.806493 | 1.066798 | 0.635895 | 1.789695 |
| NFKB* | 0.026699 | 0.828002 | 1.027058 | 0.807226 | 1.306758 |
| BCR.Shannon+ | 0.021644 | 0.888526 | 1.02188 | 0.755028 | 1.383046 |
| Indel.Neoanti+ | 0.060893 | 0.917525 | 1.062785 | 0.335662 | 3.36503 |

**Table S5**. The summary correlation coefficients of inhibitory signatures with ICR vs. MIRACLE across 28 TCGA cohorts.

| Correlation Coefficients | Min | | Median | | Mean | | Max | |
| --- | --- | --- | --- | --- | --- | --- | --- | --- |
| Signatures | ICR | Miracle | ICR | Miracle | ICR | Miracle | ICR | Miracle |
| ESTIMATE_stroma | 0.27 | -0.75 | 0.49 | 0.11 | 0.49 | 0.03 | 0.72 | 0.32 |
| EMT* | 0.26 | -0.59 | 0.45 | 0.04 | 0.46 | -0.02 | 0.68 | 0.18 |
| LAP_Helios* | 0.05 | -0.48 | 0.33 | -0.01 | 0.30 | -0.03 | 0.52 | 0.31 |
| PI3Kgamma* | 0.05 | -0.67 | 0.31 | -0.07 | 0.30 | -0.12 | 0.63 | 0.10 |
| CAF* | -0.05 | -0.29 | 0.29 | -0.01 | 0.28 | -0.03 | 0.59 | 0.22 |
| EMT_msigdb* | -0.05 | -0.79 | 0.23 | -0.17 | 0.26 | -0.21 | 0.67 | 0.01 |
| TGF.b.Resp+ | -0.07 | -0.82 | 0.19 | -0.19 | 0.23 | -0.22 | 0.64 | 0.02 |
| Des_stroma* | -0.14 | -0.66 | 0.18 | -0.18 | 0.20 | -0.21 | 0.57 | 0.03 |
